# Validity and Reliability of Wearable Sensors for Joint Angle Estimation: A Systematic Review

**DOI:** 10.3390/s19071555

**Published:** 2019-03-31

**Authors:** Isabelle Poitras, Frédérique Dupuis, Mathieu Bielmann, Alexandre Campeau-Lecours, Catherine Mercier, Laurent J. Bouyer, Jean-Sébastien Roy

**Affiliations:** 1 Centre for Interdisciplinary Research in Rehabilitation and Social Integration and Université Laval, Quebec City, QC G1M 2S8, Canada; Isabelle.poitras.2@ulaval.ca (I.P.); frederique.dupuis.1@ulaval.ca (F.D.); mathieu.bielmann.1@ulaval.ca (M.B.); Alexandre.Campeau-Lecours@gmc.ulaval.ca (A.C.-L.); Catherine.Mercier@rea.ulaval.ca (C.M.); Laurent.Bouyer@rea.ulaval.ca (L.J.B.); 2 Department of Rehabilitation, Université Laval, Quebec City, QC G1V 0A6, Canada; 3 Department of Mechanical Engineering, Université Laval, Quebec City, QC G1V 0A6, Canada

**Keywords:** criterion validity, inertial measurement unit, gold standard, joint angle, systematic review, human movement

## Abstract

Motion capture systems are recognized as the gold standard for joint angle calculation. However, studies using these systems are restricted to laboratory settings for technical reasons, which may lead to findings that are not representative of real-life context. Recently developed commercial and home-made inertial measurement sensors (M/IMU) are potentially good alternatives to the laboratory-based systems, and recent technology improvements required a synthesis of the current evidence. The aim of this systematic review was to determine the criterion validity and reliability of M/IMU for each body joint and for tasks of different levels of complexity. Five different databases were screened (Pubmed, Cinhal, Embase, Ergonomic abstract, and Compendex). Two evaluators performed independent selection, quality assessment (consensus-based standards for the selection of health measurement instruments [COSMIN] and quality appraisal tools), and data extraction. Forty-two studies were included. Reported validity varied according to task complexity (higher validity for simple tasks) and the joint evaluated (better validity for lower limb joints). More studies on reliability are needed to make stronger conclusions, as the number of studies addressing this psychometric property was limited. M/IMU should be considered as a valid tool to assess whole body range of motion, but further studies are needed to standardize technical procedures to obtain more accurate data.

## 1. Introduction

Physical rehabilitation assessments often require quantification of body movement over an extended period of time during functional or work-related tasks in order to characterize motor dysfunction, to determine task requirement, or to assess the effectiveness of an intervention. This can be performed in a laboratory setting using motion-capture systems [1], as these systems ensure high data quality and are commonly recognized as the gold standard for movement quantification [2]. However, these systems can only be used in controlled environments due to their lack of portability and to the technical expertise required, thereby limiting their potential clinical applications.

The use of wearable sensors has been suggested to overcome this lack of portability, as they are wireless, small, and light [3]. Activity, energy expenditure, and acceleration data collected by accelerometers have been shown to be valid and reliable. However, kinematic data have been shown to lack precision since they are affected by increased segment acceleration or the presence of drifts (algorithms have been developed to decrease the effect of drifting in accelerometers) [4,5,6]. Combining a gyroscope and a magnetometer with an accelerometer in a sensor, which is called an inertial measurement unit (M/IMU; sensors combining accelerometer and gyroscope [IMU] and sensors combining accelerometer, gyroscope, and magnetometer [MIMU]), has been proposed to solve this problem. Gyroscopes are used to estimate sensor orientation by integration of signals (angular velocity relative to the sensor XYZ axis) [7], accelerometers to provide a static orientation measurement relative to gravity by analyzing the acceleration signal, and magnetometers to provide heading using sensor orientation relative to Earth’s magnetic field [8]. By combining the signals of each sensor through intelligent data fusion algorithms, one can obtain a reliable estimate of M/IMU orientation. For example, accelerometers combined with magnetometers properly estimate slow-motion displacements, while the gyroscope provides better results during highly dynamic movements. Therefore, complimentary data are obtained when all three signals are captured [9]. M/IMUs provide more accurate kinematic data than accelerometers alone, while still being wireless, robust, and portable. Several commercial or home-made M/IMU systems are currently available for research and clinical applications, but as highlighted by Fong and Chan, some improvements on data processing, data logging, and fixation need to be made before these systems can be used more extensively. This could explain the growing interest in this research area during the past few years [10].

The psychometric properties, such as validity and reliability, of M/IMUs for orientation and joint kinematics estimation have been extensively studied during the past few years. Two systematic reviews have been conducted to report on the psychometric evidence relating to M/IMU validity [11,12]. The first systematic review published in 2010 concluded that M/IMUs could be a valid tool to measure range of motion, but results were variable across joints and tasks performed [8]. The other systematic review, published in 2018, focused only on the upper limb and provided only scarce details regarding the complexity of the tasks evaluated, a problem highlighted by the first systematic review as being one of the most challenging aspects for clinical evaluation. This highlights the need for a synthesis and an update for whole body kinematics. The aim of this study was therefore to conduct a systematic review of the psychometric evidence related to criterion validity and reliability of M/IMUs for the assessment of joint movement.

## 2. Materials and Methods

This systematic review follows the Preferred Reporting Items for Systematic Reviews and Meta-Analyses (PRISMA) guidelines [13] and was prospectively registered with the International Prospective Register of Systematic Reviews (PROSPERO) in August 2018 (CRD42018104775).

### 2.1. Description of the Systems

An inertial measurement unit (M/IMU) is composed of 9 sensors: 3 accelerometers, 3 gyroscopes, and 3 magnetometers.

Accelerometers: The accelerometer measures the linear acceleration in 3 orthogonal directions (XYZ). It assumes that the Z axis is aligned with gravity and gives X and Y orientations, which are useful to provide static orientation measurements. However, when sensors are moving (i.e., during active movement), the estimated orientation is biased. This is explained by the movement artifacts added to the acceleration signals, which no longer allow consideration of the Z axis as pure gravity acceleration [4]. A method often used to calculate joint kinematics is to perform a double integration of linear acceleration and a simple integration of angular velocity. This method, however, can lead to biased data by adding a drift [6]. Some computational techniques have been proposed to fix this problem such as using filters [14] or performing data realignments [15], but they are not suitable for real-time processing [16].

Gyroscopes: The gyroscopes provide angular velocity around 3 orthogonal axes (yaw, pitch, and roll; XYZ) and, theoretically, signal integration could provide an estimate of sensor orientation by assuming a known initial orientation. As it is impossible to obtain an unbiased angular velocity zero, this integration leads to a drift biasing the resulting orientation estimate [17].

Magnetometers: The magnetometers provide the orientation relative to the Earth**’**s magnetic field, which is also the sensor orientation around the Z axis. This completes the orientation calculated from accelerometers, but magnetometers are sensitive to magnetic field disturbances which can bias the sensor orientation estimate [8].

**Figure 1:**
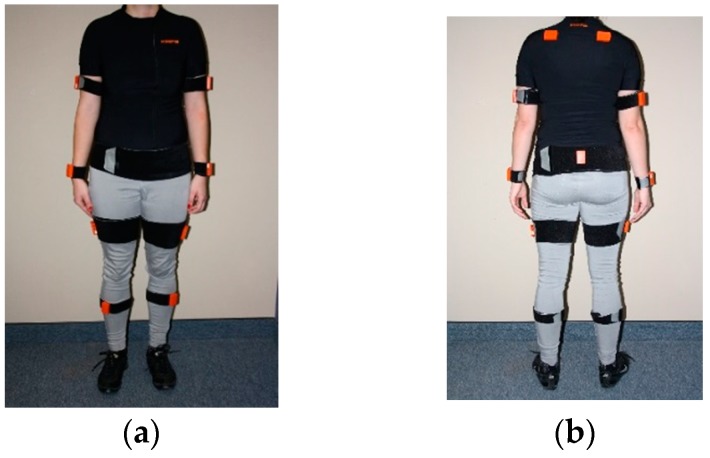
Sensors placement example, (**a**) front view, (**b**) back view.

Briefly, each sensor unit provides estimation of the orientation of body segments relative to a global reference [18,19]. Alone, each sensor provides biased information under certain circumstance, but putting them together (sensor data fusion) allows their limitations to be overcome and provides more accurate sensor orientation. Then, this can be used to calculate 3D joint angular kinematics. To achieve this, accurate M/IMU orientation has to be obtained by performing an anatomical calibration which allows an accurate joint angle calculation [20]. This procedure ensures quality of the data collected and should be carefully considered when using M/IMU systems. See Figure 1 for an example of a typical M/IMU sensor placement.

### 2.2. Data Sources

Five databases were screened (PubMed, CinAHL, Ergonomic abstract, Compendex, and EMBASE) using a combination of general keywords and specialized terms for each database. General keywords were: (“psychometric property” OR “reliability” OR “validity” OR “accuracy” OR “precision”) AND (“inertial measurement unit” OR “wearable sensors” OR “inertial sensor” OR “accelerometry”) AND (“joint angle” OR “range of motion” OR “movement” OR “kinematic”). Bibliographical references of the retrieved studies were also searched to identify additional relevant publications. Specialized keywords are presented in Table S1.

Articles were included if they: (1) presented results from human subjects, (2) included participants aged from 18 to 65 years old, (3) compared M/IMUs to a gold standard (motion-capture system for lab systems or goniometer for clinical measures) for the measurement (accelerometer, gyroscope with or without magnetometer) of the range of motion data (e.g., error of measurement, correlation coefficient, numerical value) (criterion validity) or compared data collected from the same M/IMU by two different evaluators and/or during two different sessions (reliability), (4) were written in French or English, and (5) were published after 2005. Older articles were excluded as they were not representative of current technology. Book chapters, congress abstracts, or conference proceedings, systematic reviews, and meta-analysis were excluded. Articles were not considered if they reported only data related to physical activity or energy expenditure. Titles and abstracts of the identified papers were screened independently by two reviewers (IP: Isabelle Poitras and FD: Frédérique Dupuis) to identify eligible articles. Initial data base screening was performed on 1 March, 2018 and an update was done on 15 July, 2018. A full review of the latter was performed independently by both reviewers. Any discrepancy was resolved during a consensus meeting, and a third reviewer (JSR: Jean-Sébastien Roy) was available if needed but turned out not to be required.

### 2.3. Quality Assessment

All included articles were independently critically appraised by two reviewers (IP and FD) using two different quality assessment tools: consensus-based standards for the selection of health measurement instruments (COSMIN) [21] and the critical appraisal tool by MacDermid (2008) [22]. The COSMIN grid was used to critically appraise how the psychometric properties were evaluated (reliability [box B], measurement errors [box C], and validity [box H]). The critical appraisal assessment by MacDermid was used to evaluate the methodological quality (study questions, study design, measurements, analyses, and recommendations). It gives a score out of 22. These two grids taken together allow a complete assessment of the overall article quality. Firstly, the three reviewers (IP, FD, and JSR) participated in a calibration review meeting in which each critical appraisal item was discussed. Secondly, two of the reviewers (IP and FD) independently reviewed each article. During a second meeting, every item of both grids was openly discussed until consensus on a given score was reached. If reviewers were unable to reach consensus, the third reviewer scored the article (JSR) and a discussion was performed to reach consensus.

For each grid, the score was converted into a percentage. The quality score of both tools was characterized as follows: very low (VLQ) = 0–25%, low (LQ) = 26–50%, moderate (MQ) 51–75%, and high (HQ): 76–100% [23]. Pre-consensus inter-rater agreements were calculated by using weighted Gwet’s coefficient on each individual item of the COSMIN grid. The level of agreement between reviewers was defined as: poor <0.0, slight 0.0 to 0.20, fair 0.21 to 0.40, moderate 0.41 to 0.60, substantial 0.61 to 0.80, and almost perfect 0.81 to 1.00 [24]. An intraclass correlation coefficient (ICC) was calculated on the overall score to assess inter-rater reliability for the MacDermid’s critical appraisal tool. ICC score was defined as: values <0.5 indicate poor reliability, values between 0.5 and 0.75 indicate moderate reliability, values between 0.76 and 0.9 indicate good reliability, and values >0.90 indicate excellent reliability [25].

### 2.4. Data Extractions

Each of the two reviewers performed a complete data extraction of half of the included articles and the extraction was corroborated or completed by the other reviewer. Targeted variables were extracted with a standard data extraction tool [22]. Extracted variables were: participants’ characteristics, number of participants, instrumented body part, movement performed, sensor brand, gold standard used, reliability, and validity indices. If an article presented results for more than one joint, they were treated separately to simplify data analysis.

Variables extracted for validity were: r (simple correlation coefficient), r^2^ (coefficient of determination), CMC (coefficient of multiple correlation), intraclass correlation coefficient (ICC), root mean square error (RMSE/RMS; measurement error between the reference system and M/IMU), and lower limit of agreement (LoA; total error of a measurement procedure). Variables extracted for reliability were: CMC (coefficient of multiple correlation), intraclass correlation coefficient (ICC), lower limit of agreement (LoA; total error of a measurement procedure), index of dependability (correlation coefficient), m-estimators (extremum estimators), and standard deviation/standard error of the mean (SD/SEM). ICC, r, CMC, and r^2^ were considered as poor (<0.5), moderate (between 0.5 and 0.75), good (between 0.75 and 0.9), or excellent (>0.90) [17]. Error (RMSE/RMS, LoA, m-estimators, SD, SEM) of <5° was considered as excellent and between 5 and 10° as good [12].

### 2.5. Data Analysis

Simple movements were defined as movements performed in one plane of movement (sagittal, frontal, or transverse) and complex movements were characterized as movement performed in more than one plane of movement such as walking, swimming, or sport activities. Due to the heterogeneity of protocols and data acquisition systems used in the selected articles, results of this systematic review could not be pooled in a meta-analysis. For this reason, only a descriptive synthesis of results was performed.

Four domains of concern have been selected to characterize the evidence for each joint: (1) number of studies/participants (imprecision); (2) methodological quality (risk of bias); (3) methodological and outcome similarities (indirectness); and (4) direction of results (inconsistency). Finally, the scale used to identify the level of evidence is defined as follow [26,27]:

- Strong evidence: multiple HQ studies with consistent results.
- Moderate evidence: multiple studies, including at least one HQ study, or multiple MQ or LQ studies, presenting consistent results.
- Conflicting evidence: multiple studies providing inconsistent results, regardless of the methodological quality.
- Limited evidence: multiple MQ or LQ studies with inconsistent results, or one HQ.
- Very limited evidence: only one LQ or MQ study.

## 3. Results

**Figure 2:**
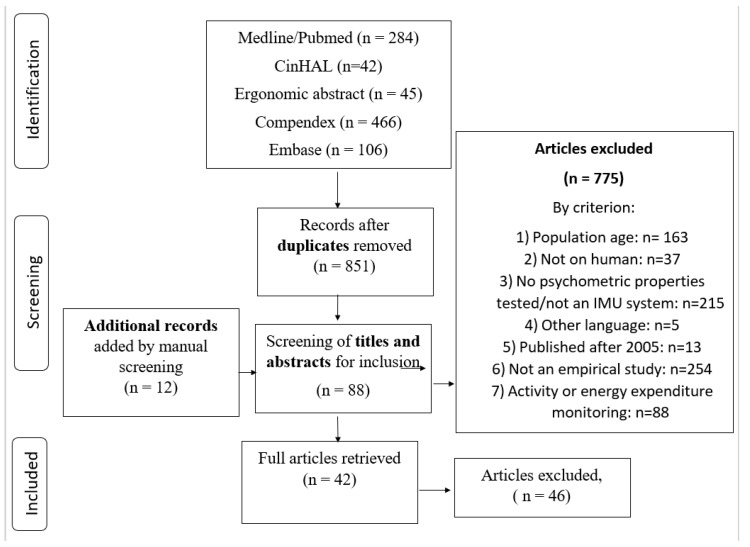
Flow chart of systematic review process.

Nine hundred and forty-three articles were retrieved. After removal of duplicates, screening of titles/abstracts, full-text analysis and manual source detection, forty-two articles were included (see Figure 2).

### 3.1. Characteristics of Studies

Thirty-nine articles addressed M/IMU validity and fifteen reported on reliability. The majority of the studies reported r and RMSE for validity (only one article used m-estimators) and ICC or CMC for reliability [28]. All of the fifteen studies assessing reliability evaluated intra-rater reliability, with four also assessing inter-rater reliability. A total of 556 subjects were tested in the included studies (508 for validity and 184 for reliability). The following body regions were investigated for validity and/or reliability: neck, shoulder, scapula, elbow, wrist, trunk, pelvis, hip, knee, and/or ankle. The knee and the hip were the most investigated joints for validity (respectively 15 and 13 studies). Reliability was only evaluated in one to three studies per joint. Across the 42 studies, the majority focused on complex movements (n = 25) as compared to simple movements (n = 17). Among the studies on complex movements, walking/running was the most frequently investigated (17/25), other movements including kicking, throwing, swimming, and sweeping a table. Ten articles reported results for various complex movements [29,30,31,32,33,34,35,36,37,38,39]. The Xsens was the most studied M/IMU system (15 articles) while other systems were investigated in three or less articles (e.g., Cuela or Physilog). See Supplementary Data Table S2 and Table S3 for detailed psychometric property results.

### 3.2. Methodological Quality

The scores on the MacDermid critical appraisal tool ranged from 41 to 92% (see Table S4) with a mean of 64.5 ± 12.9%. Eleven articles were classified as HQ studies, twenty-two as MQ, and nine as LQ. COSMIN results are presented by box (Tables S5–S7). Scores for box B (reliability) ranged from 22 to 100% with a mean of 67.4 ± 23.1%. Scores for box C (measurement error) ranged from 50 to 100% with a mean of 71.9 ± 12.8%. Scores for box H ranged from 17 to 100% with a mean of 56.4 ± 20.1%. Six studies were categorized as HQ, six as MQ, and three as LQ for reliability (combined box B and C). Seven articles were classified as HQ studies, seven as MQ, twenty-four as LQ, and one as VLQ for criterion validity. See Table 1 for detailed body evidence by body region and psychometric property. The majority of studies failed to score for sample size and for detailed inclusion/exclusion criteria, thereby partly explaining the low-quality score for both grids.

The pre-consensus inter-rater agreement between reviewers for total scores of the COSMIN grid and MacDermid’s critical appraisal tool was considered excellent (Gwet = 0.85 to 0.96 and ICC = 0.93).

### 3.3. Criterion Validity and Errors of Measurement by Body Region

#### 3.3.1. Neck

The body of evidence for the neck joint is considered as moderate (five studies, total of 44 participants [one to 20 participants/study], only one HQ study). RMSE results varied between less than 1° and 9°, showing good joint measurement validity [33,34,37,40,41]. Results are similar for the three measurement axes. Two studies reported higher variability in neck joint angle measurements (more than 7°) specifically for rotation and for two complex movements (e.g., sweeping a table, drinking), but their results were collected from only one participant in both studies. Articles presenting small RMSE used simple movements, except Pérez et al. [35] which presented validity for lifting tasks (RMSE ranged from 1.4 to 3°).

#### 3.3.2. Shoulder

The body of evidence for the shoulder joint is considered to be ‘’conflicting’’ (12 studies, total of 87 participants [one to 19 participants/study]). Despite a larger number of studies published for this joint, heterogeneity of results (RMSE between 0.2 and 64.5° and r from 0.69 to 1.00) and movements performed made it difficult to conclude the validity of using M/IMU systems for movement at this joint [31,32,33,34,35,37,38,39,42,43,44,45]. Variability increased with movement complexity, with simple movement protocols showing better concordance between studies (RMSE ranging from 1 to 21° for simple movements and from 5 to 64.5° for complex movements). Articles reported good results for flexion/extension movements (X axis; RMSE of 15° and less). Abduction/adduction movement (Z axis) showed moderate validity (RMSE of less than 20°). Validity for rotation movement in the Y axis was not consistent (high variability from 1 to 60°).

#### 3.3.3. Elbow

The body of evidence for the elbow joint is considered to be ‘’conflicting’’ (10 studies, total 71 participants [one to 19 participants/study]). RMSE and correlation coefficient results range from 0.2 to 30.6° and 0.68 to 0.99, respectively, showing good to excellent validity [28,31,32,33,34,35,37,39,43,45]. Pro-supination (r between 0.68 and 0.99) showed poorer correlations than flexion–extension (r between 0.85 and 0.99) and validity tended to decrease with increasing movement complexity (r ≥ 0.93 compared to r ≥ 0.68 for complex movements). Results should be taken with caution however, due to the heterogeneity of the reported movements (i.e., the validity was highly variable across the movements and planes studied).

#### 3.3.4. Wrist

The body of evidence for the wrist joint is considered to be ‘’conflicting’’ (6 studies, total of 51 participants [one to 19 participants/study]). RMSE and correlation coefficients varied respectively from 2.2 to 30° and from 0.62 to 0.99, showing fair to good validity [28,32,33,35,37,43]. Radial and ulnar deviation showed poorer results than any other movement (RMSE ranging from 3 to 30° for ulnar deviation compared to 3 to 20° for flexion), regardless of the movements performed. Contradictory results were given from one study using home-made sensors which presented poor results for all of the movements performed (RMSE higher than 30° and r as low as 0.62) [43].

#### 3.3.5. Trunk

The body of evidence for the trunk is considered to be strong (11 studies for the lumbar and thoracic spine, total of 167 participants [one to 38 participants/study], four HQ studies). Validity was similar across all movement planes and axes [34,36,37,38,41,46,47,48,49,50,51]. Two studies reported conflicting results for this joint, but they are also the two with the poorest methodological quality (RMSE between 1.4 and 26.2° and r as low as 0.05) [34,49]. RMSE and correlation coefficients varied respectively from 1.8 to 5.9° and from 0.72 to 0.99 when excluding data from these two articles, showing good to excellent validity.

#### 3.3.6. Pelvis

The body of evidence for the pelvis joint (tilt or obliquity) is considered to be strong (6 studies, total of 95 participants [six to 30 Participants/Study], three HQ studies). RMSE and correlation coefficients varied respectively from 0.4 to 8.8° and 0.84 to 1.00, showing good to excellent validity [30,36,38,41,42,52]. Results were similar across movement planes and axes. Two articles reported poorer results (RMSE from 5.0 to 8.8°), but only for complex movements (milking cluster attachment task/sit-to-stand/block step).

#### 3.3.7. Hip

The body of evidence for the hip joint is characterized as strong (13 studies, total of 195 participants [three to 30 participants/study], two HQ studies and eight MQ studies with similar results). RMSE and correlation coefficients varied respectively from 0.2 to 9.3° and 0.53 to 1.00, showing fair to excellent validity [5,30,37,41,42,46,53,54,55,56,57,58,59]. Hip rotation and movements performed in the transverse plane (Y axis) showed higher RMSE and lower correlations (RMSE as high as 11.8° and r as low as 0.35).

#### 3.3.8. Knee

The body of evidence for the knee joint is considered to be strong (15 studies, total of 228 participants [three to 38 participants/study], three HQ and nine MQ studies). RMSE and coefficient correlation varied respectively from 1 to 11.5° and 0.4 to 1.00 [5,30,37,41,43,48,50,53,54,55,57,58,59,60,61]. Knee abduction/adduction, which are movements with very small range of motion (ROM), were less valid (RMSE higher than 5°) than knee flexion and extension, which presented high validity (majority of studies with r ≥ 0.8).

#### 3.3.9. Ankle

The body of evidence for the ankle joint is considered to be strong (11 studies, total of 141 participants [three to 26 participants/study], three HQ and nine MQ studies). RMSE and correlation coefficients varied respectively from 0.4 to 18.8° and 0.33 to 0.99 [29,37,41,43,53,54,55,57,59,62,63]. RMSE results increased with movement speed (different walking speed vs. running; RMSE of 1.2 to 2.0 for a slow walking speed to 15.8° for running). Also, rotation and inversion/eversion showed poorer validity than dorsiflexion/plantar flexion, especially for small ranges of movement (RMSE as high as 18.8 for rotation and 11.2 for inversion/eversion).

### 3.4. Reliability by Joint

#### 3.4.1. Neck

The body of evidence for the neck is considered to be moderate (three studies, total of 48 participants [10 to 20 participants/study], one HQ study and two MQ). Standard error of the mean (SEM) and ICC for inter-rater reliability ranged respectively from 1.2 to 9.4° and from 0.64 to 0.99 [64,65,66]. Cervical rotation showed poorer ICC (two studies with ICC < 0.8), while flexion/extension and lateral flexion showed excellent reliability (ICC ≥ 0.8).

#### 3.4.2. Shoulder

The body of evidence for the shoulder is considered to be limited (two studies, total of 30 participants (10 to 20 participants/study], one HQ study and one MQ). SEM and ICC for inter-raterreliability ranged respectively from 4.2 to 7.8° and 0.71 to 0.99 [28,66]. Flexion/extension movements showed higher reliability than abduction and rotation (RMSE: flexion–extension: 5.9–7.2°; abduction-adduction: 4.2–5.7°; rotation: 6.6–7.8°; r: flexion–extension: 0.96-0.99; abduction–adduction: 0.72–0.91; rotation: 0.72–0.87).

#### 3.4.3. Scapula

The body of evidence for the scapula is considered to be limited (one study, total of 20 participants, one HQ study). Inter-rater reliability is considered as fair to good for scapular retraction–protraction and scapular mediolateral rotation (r = 0.59 to 0.91), but as poor to fair for anterior–posterior tilt (r = 0.32 to 0.87) [67].

#### 3.4.4. Elbow

The body of evidence for the elbow is considered to be conflicting as the two studies included presented inconsistent results (two studies, total of 30 participants [10 to 20 participants/study]). One study showed excellent reliability in every movement direction (CMC ≥ 0.9) and the other presented poor to good reliability (ICC = 0.2 to 0.77) [28,66].

#### 3.4.5. Wrist

The body of evidence for the wrist is considered to be limited (two studies, total of 30 participants [10 to 20 participants/study], one HQ study and one MQ). Both studies presented good to excellent intra-rater reliability for all joint movements (CMC and ICC between 0.79 to 0.96) [28,66].

#### 3.4.6. Trunk

The body of evidence for the trunk is considered to be moderate (three studies, total of 63 participants [19 to 24 participants/study], two HQ and one MQ study). Results from these three studies are consistent and demonstrate good to excellent intra-rater reliability (ICC ≥ 0.8 except for coronal plane which is fair to excellent (0.67 to 0.93)) [46,66,68].

#### 3.4.7. Pelvis

The body of evidence for the pelvis is considered to be moderate (one study, total of one participant, one HQ study). All pelvis movements showed good reliability with an absolute error of less than 1.2° [42].

#### 3.4.8. Hip

The body of evidence for the hip is considered to be moderate (three studies, total of 70 participants [20 to 26 participants/study], one HQ and two MQ study). Intra-rater reliability is fair to excellent and inter-rater reliability varied across movements performed (good for walking [ICC of 0.4 to 0.74] and poor for jumping [ICC ≤ 0.39] [46,53,66]).

#### 3.4.9. Knee

The body of evidence for the knee is considered to be moderate (three studies, total of 56 participants [10 to 26 participants/study], one HQ and two MQ study). Intra-rater reliability was fair to excellent and inter-rater reliability varied across movements performed (good for walking [ICC from 0.4 to 0.74] and poor for jumping [ICC ≤ 0.39] [45,53,59]).

#### 3.4.10. Ankle

The body of evidence for the ankle is considered to be moderate (three studies, total of 52 participants [12 to 26 participants/study], one HQ and two MQ study). Intra- and inter-rater reliability varied across movements complexity (better for walking [ICC of 0.6 to 0.95] than jumping [ICC of 0.39 to 0.99] or playing soccer [ICC of 0.28 to 0.79] [29,53,62]).

## 4. Discussion

This systematic review of 42 studies assessed available evidence on psychometric properties of the use of M/IMUs to quantify joint movement. Regarding validity, M/IMUs appear to provide a suitable alternative to motion-capture systems especially for flexion/extension movements at the lower limb joints during simple movements. This systematic review has provided an overview of current knowledge on the validity and reliability of the M/IMU system for the whole body, as an update to a similar past review from Cuesta-Vargas et al. published in 2010 [12] and complements a recent review on the upper limb that focused more on the technical aspects of M/IMU use (calibration, type of sensors) than on clinical applications (movement complexity, more details on specific movement at each joint) [11]. The review from Cuesta-Vargas et al. concluded that validity depended on the joint studied and movement performed. However, they did not report detailed results for each joint separately and only few comments were made on movement complexity [12]. More specifically, they reported that increasing movement complexity decreases the validity, but most of the cited studies they reported used only simple planar movement protocols such as isolated flexion–extensions, which limited the generalizability of their conclusions. Regarding Walmsley et al.’s review, their search was limited to the upper limb [11]. Their results for the upper limbs are in agreement with those presented here. In addition, they included conference proceedings, articles comparing M/IMUs with robotic arms, and studies presenting data without comparison to a “gold standard”. The increasing number of studies in recent years that used complex movements to assess M/IMU validity and the focus made on the upper limb joint partially explain the different conclusions between these two systematic reviews and the present work [12]. Indeed, ecological tasks related to activities of daily living generally require complex movements, the use of both upper and lower limb joints, movements in more than one plane, and irregular movement speed, which all drastically affect system performance (e.g., walking at different speed vs. running [62,63]). For example, studies reporting the worst validity assessed sporting tasks or activities of daily living such as sweeping a table [32,34]. Proper calibration (proper alignment of the M/IMU axes with the anatomical segment axes) is another important factor contributing to better reliability, as different calibration protocols may result in substantially different results [28]. As the initial calibration is the reference to calculate joint angulation during body movement, a poor calibration in which sensors are not aligned with the body segment could interfere with data quality. It was not possible in this systematic review to reach a conclusion on the best calibration procedure for a given movement/context, as many studies did not identify or sufficiently described their calibration procedure.

The previous systematic review on whole body kinematics reported better results for upper limb joints (elbow, wrist, shoulder) than lower limb joints [12]. This is opposite to our results. We considered evidence related to the validity of shoulder, elbow, and wrist movements to be conflicting, particularly when participants were involved in complex movements or when the joint rotation around the Y axis was targeted. Functional upper limb tasks often require performing complex movements around two or three axes (X, Y, Z), which can account for the variability of results. In contrast, lower limb and trunk movements are mostly performed in closed chains and in one plane of movement, providing more consistent results, except for movements with smaller ROM (e.g., knee abduction/adduction or ankle internal/external rotation). Also, as demonstrated by Robert-Lachaine et al. [37], the choice of the biomechanical model used to convert M/IMU orientation data into anatomically meaningful information will also influence validity differently depending on the joint studied. Their results showed that biomechanical models used for the upper limbs are more likely to add variability, leading to larger RMSE. This partly explains the large variability observed at the shoulder joint (RMSE as high as 40° in some articles). According to Robert-Lachaine et al., shoulder rotation is also more influenced by the biomechanical model than shoulder flexion–extension or abduction/adduction. Another important aspect to consider, as discussed in the Materials and Methods section, is that the weaknesses of each sensor in the estimation of joint kinematics could have separately impacted the accuracy/reliability of M/IMUs as sensor fusion does not fully compensate for all sensors limitations [9].

It is important to note here that, recently, several studies have questioned the validity and accuracy of camera-based motion capture systems for certain joints and for complex movements [46,54]. Indeed, errors ranging from 5 to 10° when compared to goniometer, inclinometer, and radiostereometric measurements have been evidenced (same error range as M/IMU systems). Interestingly, and similar to M/IMUs, errors reported for camera-based motion capture systems varied across movement planes and joints. Caution must therefore be taken when using these systems as a gold standard for validating M/IMUs.

The validity of motion capture and M/IMUs for trunk and lower limb joints are similar except for the rotation component, suggesting that M/IMUs could be a good alternative to motion capture systems for movement measurements in the sagittal and frontal planes, but not in the transverse plane. For the upper limbs, flexion/extension movements should be considered valid in every context. However, the complexity of the movement leads to more variability in abduction/adduction, which needs to be considered. For rotation, more studies are required to identify the best approach for the most accurate data. In clinical settings, M/IMUs should be considered as an alternative tool to inclinometers and goniometers, as they provide real-time data in functional tasks within the same error range as these more classical measurement devices (M/IMU: 0.7 to 15°; inclinometer and goniometer: 0.21 to 18°) for lower limb, trunk, wrist, and elbow joints. However, more time and technical resources must be allowed for patient assessment with this technology until the system becomes more user friendly.

Regarding reliability, the small number of studies for each joint did not allow us to make strong conclusions. As mentioned above for validity, reliability is better for movements in the sagittal and frontal planes than in the transverse plane. However, most of the included studies only assessed reliability during simple planar movements and locomotor tasks. Here also, more complex movements would probably show less reliable results. The different types of M/IMU gave similar reliability results (e.g., Xsens, Cuela, and Gait-up Physilog) regardless of the joint studied. Moreover, the absolute error reported for M/IMU ranged from 0.3° to 9.9° which is better than the goniometer and inclinometer reliability (inclinometer: 1° to 32°; goniometer: 1° to 45°) [69]. Reliability results are therefore promising, but more high-quality studies are needed to be able to reach stronger conclusions, especially for complex movements.

Rehabilitation research and health care services could benefit from M/IMUs as they provide valid data to assess range of motion and joint orientation. The present systematic review has focused on healthy subjects to reduce the variability in reported data, but M/IMUs need to ultimately benefit pathological populations and clinicians by guiding clinical decision making (e.g., quantify deficits and determine progress in time) [70]. As the validity has been demonstrated in a majority of body joints for healthy subjects, it opens the possibility to assess psychometric properties in pathological populations. However, special considerations will be needed in pathological populations as most of the calibration procedures require specific posture or movement, which could be challenging for some populations (e.g., cerebral palsy). Also, to be clinically useful these systems should be validated in ecological settings which could prove to be difficult considering the changing environment of the real world and the need for an established gold standard outside of the laboratory settings [8].

Limitations of this research include that the validity and reliability results presented in this systematic review represented the use of M/IMU in controlled environments as all the included studies were performed in laboratory settings. Therefore, they cannot be considered as directly representing M/IMU performance during real-life/outside of laboratory use. More studies in work and home environments need to be performed in order to generalize the findings. In addition, the lack of standardization in protocols such as the use of different biomechanical models for the same joint or of different calibration procedures and sensor placements makes it impossible to identify the main contributing factor to measurement errors. This highlights the need for specific guidelines and standardization for M/IMU, similar to the Seniam guidelines [71] for electromyography, as they could drastically reduce variability between studies. Furthermore, despite the high number of studies included in this review (42 articles), there are only twelve high quality studies, which restricted conclusions. However, the majority of studies had the same limitations: small sample sizes without justification, targeting only one psychometric property, and lack of description of inclusion/exclusion criteria. These factors have lowered study quality but did not interfere with precision of the reported data.

Most of the studies included in this review are of moderate to high quality, suggesting a low risk of bias in conclusions. The overall quality of all articles was reduced by the small sample sizes (see above). Only two articles had a good score on this criterion, but based mainly on the fact that a justification was provided. The sample size used in included studies ranged from one to 38 participants. This is below the recommended sample size for this type of study (50 participants). This could lead to misinterpretation [21]. To lower this risk of bias, we gave less impact to studies performed on only one participant.

Another important risk of bias is the heterogeneity of movements studied across the 42 articles, which did not allow us to perform quantitative analysis. The complexity of the movements assessed varied widely between studies and, as mentioned, M/IMU systems have lower validity in complex movements than in simple movements.

## 5. Conclusions

In conclusion, results from this systematic review suggest that M/IMUs are an appropriate alternative to motion-capture based systems to study human movement. However, as a restricted number of articles have been conducted on M/IMU reliability it is not possible to reach a conclusion on the best specific procedures (e.g., the calibration) to ensure reliable data. More studies in ecological environments are also needed before using M/IMU extensively outside of the laboratory. The main advantage of M/IMUs is that they can provide accurate data at a lower cost than a motion-capture system. However, M/IMU users should consider movement complexity, sensor placement, joint studied, biomechanical models used, and calibration procedure, before drawing specific conclusions. Further studies are needed to standardize data acquisition protocols with these devices to allow a meta-analysis of the results.

## Figures and Tables

**Table 1:** Synthesis of overall quality and body of evidence by joint.

Joint	Validity: Number of Articles	Reliability: Number of Articles	COSMIN Quality Evidence – Validity	COSMIN Quality Evidence – Reliability	MacDermid Quality Evidence – Validity	MacDermid Quality Evidence – Reliability	Body of Evidence – Validity	Body of Evidence – Reliability
**Neck**	5	3	1 MQ 3 LQ 1 VLQ	1 HQ 1 MQ 1 LQ	1 HQ 2 MQ 2 LQ	1 HQ 2 MQ	Moderate	Moderate
**Scapula**	0	1	N/A	1 HQ	N/A	1 HQ	N/A	Limited
**Shoulder**	12	2	1 HQ 3 MQ 7 LQ 1 VLQ	1 MQ 1 LQ	1 HQ 7 MQ 4 LQ	1 HQ 1 MQ	Conflicting evidence	Moderate
**Elbow**	10	2	1 HQ 3 MQ 5 LQ 1 LQ	1 MQ 1 LQ	1 HQ 5 MQ 4 LQ	1 HQ 1 MQ	Conflicting evidence	Conflicting evidence
**Wrist**	6	2	2 MQ 4 LQ	1 MQ 1 LQ	1 HQ 3 MQ 2 LQ	1 HQ 1 MQ	Moderate	Moderate
**Trunk**	11	3	1 HQ 4 MQ 5 LQ 1 VLQ	1 MQ 2 LQ	4 HQ 6 MQ 1 LQ	2 HQ 1 MQ	Strong	Moderate
**Pelvis**	6	1	4 MQ 2 LQ	1 HQ	3 HQ 3 MQ	1 HQ	Strong	Limited
**Hip**	13	3	2 HQ 3 MQ 8 LQ	1 HQ 1 MQ 1 LQ	2 HQ 8 MQ 3 LQ	1 HQ 2 MQ	Strong	Moderate
**Knee**	15	3	2 HQ 3 MQ 10 LQ	1 HQ 2 MQ	3 HQ 9 MQ 3 LQ	1 HQ 2 MQ	Strong	Moderate
**Ankle**	11	3	3 HQ 2 MQ 6 LQ	3 HQ	3 HQ 6 MQ 2 LQ	2 HQ 1 MQ	Strong	Moderate

HQ = high quality; MQ = moderate quality; LQ = low quality; VLQ = very low quality; N/A = not applicable.

## Supplementary Materials

The following are available online at https://www.mdpi.com/1424-8220/19/7/1555/s1: Table S1: Specialized keywords used for each database search; Table S2: Synthesis of criterion validity results; Table S3: Synthesis of reliability results; Table S4: Assessment of methodological quality by an appraisal quality tool (MacDermid et al., 2008); Table S5: Assessment of studies examining measurement errors (absolute measures) using the COSMIN checklist (box B); Table S6: Assessment of studies examining measurement errors (absolute measures) using the COSMIN checklist (box C); Table S7: Assessment of studies examining criterion validity using the COSMIN checklist (box H)

## Trial Registration

This systematic review was registered with PROSPERO (CRD42018104775).
