# Supplementary material for: Validity and Reliability of Wearable Sensors for Joint Angle Estimation: A Systematic Review
Source: Sensors (Basel). 2019 Mar 31;19(7):1555. doi: 10.3390/s19071555 (PMC6479822; doi:10.3390/s19071555)
Supplement: Supplementary file 1 [file sensors-19-01555-s001.pdf]

Table S1 : Specialized keywords used for each database search.

	**Psychometric properties**		**Data Acquisition Systems**		**Movements**
**General keywords**	psychometric property OR reliability OR validity		inertial measurement unit OR wearable sensor OR inertial sensor OR accelerometry		joint angle OR range of motion OR movement OR kinematic
**MeSH (Pubmed)**	psychometrics OR reproducibility of results	**AND**	physiology/instrumentation OR accelerometry	**AND**	biomechanical phenomena OR range of motion, articular OR movement
**CinAHL descriptors**	reliability and validity		wearable sensors OR wearable technology		kinematic OR range of motion
**Thesaurus (Ergonomic abstract)**	test validity OR reliability		wearable technology		range of motion).
**Ei mainheading (Compendex)**	reliability OR reliability analysis		biomedical equipment OR wireless sensor networks OR wearable sensors)		joint anatomy
**Emtree (Embase)**	validity OR reliability		electronic sensor		range of motion

Table S2 : Synthesis of criterion validity results.

Authors	n	IMU used	Motion capture system used	Task	Validity
**Knee**					
Al-Amri & al.	26	Xsens MVN BIOMECH (Xsens technologies B.V., Enschede, the Netherlands)	Vicon Motion Systems (Oxford Metrics Ltd, Oxford, UK)	Walking<br><br>Jump<br><br>Squat	• Sagittal plane: CMC > 0.9 et r^2 > 0.8<br>• Frontal and transverse plan: r^2 = 0.4-0.8
Bergmann, J. H. & al.	14	Xsens MVN BIOMECH (Xsens technologies B.V., Enschede, the Netherlands)	Codamotion (Charnwood Dynamics, Leicestershire, UK)	Climbing steps	Pearson Coefficient (r)/ RMSE (°) (mean±SD): 0.98 ±0.05 / 4 ± 3
Blair, S & al.	30	Xsens MVN BIOMECH (Xsens technologies B.V., Enschede, the Netherlands)	Vicon Motion Systems (Oxford Metrics Ltd, Oxford, UK)	Kicking tasks	**Mean difference ± 90% CL**<br>Australian football: 0.1 ± 1.1<br>Soccer: 0.3 ± 1.1<br>Rugby: 0.2 ± 1.1
Cloete, T. & al.	8	Xsens MVN BIOMECH (Xsens technologies B.V., Enschede, the Netherlands))	Vicon Motion Systems (Oxford Metrics Ltd, Oxford, UK)	Walking	**Flexion/extension**<br>RMSE (°) (SD) / R (mean + SD)<br>Right: 7.61 (4.47)/ 0.92 (0.08)<br>Left: 9.33 (5.45)/ 0.85 (0.22)<br>**Valgus/varus**<br>RMSE (°) (SD) / R (mean + SD)<br>Right: 10.22 (4.71) /0.26 (0.48)<br>Left: 11.46 (6.90)/ 0.12 (0.41)<br>**Rotation:**<br>RMSE (°) (SD) / R (mean + SD)<br>Right: 6.44 (2.83)/ 0.25 (0.44)<br>Left: 8.18 (4.28)/ 0.24 (0.40)
Dejnabadi, H. & al.	8	Accelerometer chips ADXL202/210 and yaw rate gyro chips ADXRS150/300	Ultrasound-based motion measurement (Zebris,D)	Walking	Average RMSE error: 1 °<br>Mean: 0.5°<br>SD: 0.8°<br>CMC: 0.999
Dowling, A. V. & al.	38	Inertial measurement units (PhysilogVR , BioAGM, CH)	Qualisys (Medical, Gothenburg, SE)	2-drop jumps with a unilateral support landing	(**R** / accuracy °/precision °):<br>Land prep: **0.97**/-3.7/2.4<br>Contact: **0.92**/-4.7/3.4<br>Max stance: **0.93**/-2.4/3.9<br>Difference: **0.94/**2.3/3.9
Favre, J. & al.	10	IMUs (ADXRS and ADXL) (PhysilogVR , BioAGM, CH)	Libertys (Polhemus, USA)	Maximal active ROM<br><br>Walking (30m)	**μ /RMS /CC (mean ±SD)**<br>Flexion extension: -1.0± 1.0/ 1.5±0.4/1.00±0.00<br>Abduction/adduction: 0.0 ±0.6/1.7±0.5/0.86±0.08<br>Internal-external rotation: 3.4±2.7/ 1.6±0.5/

					0.95±0.03
Jaysrichai T. & al.	10	IMU sensors (SparkFun Electronics, Niwot, CO, USA)	Qualisys (Medical, Gothenburg, SE)	Knee flexion<br><br>Hip and knee flexion test<br><br>Forward step test<br><br>Leg abduction test<br><br>Walking (normal speed)	**RMS(Min-Max) (°)/ ICC (Min-Max):**<br>**Knee flexion**<br>right: 1.5-3.6/0.99-1.00<br>left: 2.5-4.7/0.97-1.00<br>**Hip and knee flexion test**<br>Right: 1.6-3.9/ 0.99-0.99<br>left: 2.6- 5.4/ 0.97-0.99<br>**Forward step test**<br>right: 2.4-4.2/0.88-0.96<br>left: 1.9-5.2/0.85-0.99<br>**Leg abduction test**<br>right :1.1-4.0/-0.26-0.86<br>left: 1.4-2.1/-0.27-0.87<br>**Walk**<br>right: 2.7-5.3/0.84-0.98<br>left: 2.3-5.1/0.84-0.98
Kumar Y. & al.	19	Home-made sensors systems	Goniometer	Maximal active ROM	r^2<br>0.82 (flexion only)<br>Lower extremity ± 10°
Leardini A. & al.	17	Riablo™ (CoRehab, Trento, Italy)<br><br>*An adaptive system, comprised of several IMU*	Vicon Motion Systems (Oxford Metrics Ltd, Oxford, UK)	1. Knee flexion against gravity in single leg up-right posture<br><br>2. Knee extension against gravity from the chair<br><br>3. Lunge<br><br>4. Squat	**RMSE - Mean ± SD (°)**<br>1. 3.9 ± 0.7<br>2. 3.8 ± 0.8<br>3. 4.5 ± 1.3<br>4. 5.0 ± 1.2
Lebel K. & al.	20	IGS-180 (synertial) (model OSv3 also called OS3D, Inertial Labs)	Vicon Motion Systems (Oxford Metrics Ltd, Oxford, UK)	Sit-to-stand<br><br>Walking<br><br>Turning	**RMSD (°)/Err peak(°)/CMC**<br>Sit-to-stand: 1.3 / 3.2 / 0.983<br>Walk: 2.4 / 5.6 / 0.990<br>Turn: 2.4 / 4.9 / 1.000
Robert-Lachaine X. & al.	12	Xsens MVN BIOMECH (Xsens technologies B.V., Enschede, the Netherlands)	Optotrak system (Northern Digital Inc., Ontario, Canada)	Handling tasks and simple movements	**RMSE/CMC/ /LoA**<br>Z= 2.3/0.99/ -0.1±4.4<br>X= 1.9/0.91/0.6±2.8<br>Y= 3.3/0.97/0.4 ±5.5
Saito, H. & al.	3	Wireless sensors<br>(WAA-006, Wireless Technologies)	Optotrak system (Northern Digital Inc., Ontario, Canada)	Walking (3 different speeds)	<u>**Without Kalman Filter**</u><br>**RMSE (SD)**<br>5.73 (2.15)<br>***R (SD)***<br>0.976 (0.011)

					**With Kalman Filter**<br>**RMSE (SD)**<br>3.33 (0.80)<br>***R* (SD)**<br>0.985 (0.008)
Takeda, R. & al.	3	Tri-axial acceleration sensor (H34C, Hitachi Metals, Ltd.) and three gyro sensors (ENC-03M, muRata Manufacturing Co., Ltd.)	DIPP-Motion Pro (Ditect Co., Ltd.)	Walking (5m)	**RMSE** = 6.79<br>**Absolute deviation** (AD) = 4.65<br>**Correlation coefficient** (CC) = 0.92
Zhang, JT & al.	10	Xsens MVN BIOMECH (Xsens technologies B.V., Enschede, the Netherlands)	Optotrak system (Northern Digital Inc., Ontario, Canada)	Walking<br><br>Stair descent<br><br>Stair ascent	ROM/error (°)/CMC (only in walking)<br>**Walking:** Flexion-extension= 0.8 ± 2.61 / 1.87 ±1.27/ 0.99<br>Abduction-adduction = 1.45± 10.81/ 5.09 ±4.18 / 0.71<br>Internal-external rotation = 0.03 ± 4.69 / 2.7 ± 2.2 / 0.88<br>**Stair descent:** Flexion-extension= 0.41 ± 1.59 / 1.99 ±1.07<br>Abduction-adduction= 0.65± 9.88/ 5.53 ±3.78<br>Internal-external rotation = 0.1 ± 8.53 / 3.66 ± 3.62<br>**Stair ascent:** Flexion-extension= 0.11 ± 0.68 / 1.71 ±1.45<br>Abduction-adduction = 0.79± 8.16/ 4.68 ±3.76<br>Internal-external rotation = 1.65 ± 7.08 / 5.15 ± 3.08
**Hip**					
Al-Amri & al.	26	Xsens MVN BIOMECH (Xsens technologies B.V., Enschede, the Netherlands)	Vicon Motion Systems (Oxford Metrics Ltd, Oxford, UK)	Walking<br><br>Jump<br><br>Squat	• Sagittal plan: CMC > 0.9 et $r^2 > 0.8$<br>• Transverse hip: poor
Bauer, C. M. & al.	22	IMUs<br>(Valedo_ User Manual, Hocoma AG)	Vicon Motion Systems (Oxford Metrics Ltd, Oxford, UK)	Maximal active ROM	**r^2 / RMSE**<br>Flexion= 0.99 ± 0.01 / 6.1 ±2.7<br>Extension = 0.94 ±0.09 / 5.6 ± 4.1<br>Lateral flexion right = 0.87 ± 0.21 / 1.1 ± 0.7<br>Lateral flexion left = 0.85 ± 0.2/ 1.1 ± 0.7
Bergmann, J. H. & al.	14	Xsens MVN BIOMECH (Xsens technologies B.V., Enschede, the Netherlands)	Codamotion (Charnwood Dynamics, Leicestershire, UK)	Climbing steps	Pearson Coefficient/ RMSE (°) (mean±SD) : 0.96±0.06 / 5±3
Blair, S & al.	30	Xsens MVN BIOMECH (Xsens technologies B.V., Enschede, the Netherlands)	Vicon Motion Systems (Oxford Metrics Ltd, Oxford, UK)	Kicking tasks	**Mean difference ± 90% CL**<br>Australian football: -1.4 ± 0.3<br>Soccer: -1.7 ± 0.3

					Rugby: -1.5 ± 0.3
Cloete, T. & al.	8	MOVEN inertial motion-capture system (Xsens technologies B.V., Enschede, the Netherlands)	Vicon Motion Systems (Oxford Metrics Ltd, Oxford, UK)	Walking	**Flexion/extension**<br>RMSE (°) (SD) / R (mean + SD)<br>**Right:** 5.71 (3.18) /0.94 (0.08)<br>**Left:** 5.91 (4.36) / 0.94 (0.07)<br>**Abduction/adduction**<br>RMSE (°) (SD) / R (mean + SD)<br>**Right:** 7.93 (4.53) /0.53(0.45)<br>**Left:** 6.75 (5.77) / 0.57 (0.34)<br>**Rotation**<br>RMSE (°) (SD) / R (mean + SD)<br>**Right:** 6.49 (3.16) /0.72 (0.1)<br>**Left:** 9.26 (6.63)/ 0.35 (0.29)
Dejnabadi, H. & al.	8	Accelerometer chips ADXL202/210 and yaw rate gyro chips ADXRS150/300	Ultrasound-based motion measurement (Zebris,D)	Walking	Average RMS error: 0.6 °<br>Mean: 0.1°<br>SD: 1.1°<br>CMC: 0.998
Kumar Y. & al.	19	Home-made sensors systems	Goniometer	Maximal active ROM	r^2<br>0.69 to 0.93 (abduction= 0.93, adduction=0.82, extension=0.82, flexion=0.90, internal rotation=0.79, external rotation= 0.69)<br>Lower extremity ± 10° (except for adduction, extension and flexion at the hip = ±20°)
Lebel K. & al.	20	IGS-180 (synertial) (model OSv3 also called OS3D, Inertial Labs)	Vicon Motion Systems (Oxford Metrics Ltd, Oxford, UK)	Sit-to-stand<br><br>Walking<br><br>Turning	**RMSD (°)/Err peak(°)/CMC**<br>Sit-to-stand: 0.9/ 2.4/ 0.999<br>Walk: 1.6/ 4.4/ 0.981<br>Turn: 1.3/ 3.3/ 1.000
Robert-Lachaine X. & al.	12	Xsens MVN BIOMECH (Xsens technologies B.V., Enschede, the Netherlands))	Optotrak system (Northern Digital Inc., Ontario, Canada)	Handling tasks and simple movements	**RMSE/CMC /LoA**<br>Z= 2.2/1.00/ -0.3±4.1<br>X= 2.3/0.97/0.2±3.7<br>Y= 3.5/0.95/-0.6±5.6
Saber-Sheikh, K. & al.	20	Xsens MTx sensors (Xsens technologies B.V., Enschede, the Netherlands)	Fastrak system (Polhemus, 2007)	Walking	**RMSE**<br>Mean difference: -0.05 (SD 0.77)<br>X: -0.69 (SD 0.90); Y: -0.4 (SD 1.05); Z: -0.28 (SD 1.63)
Saito, H. & al.	3	Wireless sensors (WAA-006, Wireless Technologies)	Optotrak system (Northern Digital Inc., Ontario, Canada)	Walking (3 different speeds)	**Without Kalman Filter**<br>**RMSE (SD)**<br>7.03 (3.95)<br>***R (SD)***<br>0.965 (0.019)<br><br>**With Kalman Filter**<br>**RMSE (SD)**

					3.55 (1.37)<br>***R (SD)***<br>0.989 (0.005)
Takeda, R. & al.	3	Tri-axial acceleration sensor (H34C, Hitachi Metals, Ltd.) and three gyro sensors (ENC-03M, muRata Manufacturing Co., Ltd.)	Vicon Motion Systems (Oxford Metrics Ltd, Oxford, UK)	Walking (5m)	**Flex-Ext**<br>RMSE = 8.72<br>Absolute deviation (AD) = 6.57<br>Correlation coefficient (CC) = 0.88<br><br>**ABD-ADD**<br><br>RMSE = 4.96<br>Absolute deviation (AD) = 3.30<br>Correlation coefficient (CC) = 0.72
Zhang, JT & al.	10	Xsens MVN BIOMECH (Xsens technologies B.V., Enschede, the Netherlands)	Optotrak system (Northern Digital Inc., Ontario, Canada)	Walking<br><br>Stair descent<br><br>Stair ascent	ROM/error (°)/CMC (only in walking)<br>**Walking:** Flexion-extension= 2.42 ± 3.83 / 2.47 ±1.6 /0.99<br>Abduction-adduction = 5.37± 5.56/4.83±3.16 /0.39<br>Internal-external rotation = 0.04 ± 4.38 / 3.02 ± 1.64 /0.96<br>**Stair descent:** Flexion-extension= 1.32 ± 2.87 / 1.9 ±1.24<br>Abduction-adduction = 11.78± 6.09/2.08 ±2.17<br>Internal-external rotation = 0.37 ± 3.29 / 1.38 ± 1.08<br>**Stair ascent:** Flexion-extension= 1.95 ± 3.73 / 2.41 ±1.73<br>Abduction-adduction = 3.17± 3.02/3.57 ±2.54<br>Internal-external rotation = 0.00 ± 2.23 / 2.32 ± 1.35
**Ankle**					
Akins, J. S. & al.	12	MARG sensors (X-IMU, x-io Technologies Limited, UK).	Vicon Motion Systems (Oxford Metrics Ltd, Oxford, UK)	Soccer field maneuvers	**Correlation coefficient/ RMSE***<br>Plantar-dorsiflexion = 0.935-0.975/1.64-3.595<br>Eversion-inversion = -0.74 – 0.327 / 2.857-5.175<br>Internal-external rotation =0.545-0.654/8.358-10.375<br>***only initial contact data are reported, for more details refer to article**
Al-Amri & al.	26	MTw2 trackers (Xsens technologies B.V., Enschede, the Netherlands)	Vicon Motion Systems (Oxford Metrics Ltd, Oxford, UK)	Walking<br><br>Jump	• Sagittal plan: CMC > 0.9 et $r^2 > 0.8$<br>• Frontal plan ankle (walking): poor

				Squat	
Bergmann, J. H. & al.	14	Xsens MVN BIOMECH (Xsens technologies B.V., Enschede, the Netherlands)	Codamotion (Charnwood Dynamics, Leicestershire, UK)	Climbing steps	Pearson Coefficient/ RMSE (°) (mean±SD):<br>0.93 0.05/ 4±2
Cloete, T. & al.	8	Xsens MVN BIOMECH (Xsens technologies B.V., Enschede, the Netherlands)	Vicon Motion Systems (Oxford Metrics Ltd, Oxford, UK)	Walking	**Plantar / dorsi flexion**<br>RMSE (°) (SD) / R (mean + SD)<br>Right: 11.6 (4.27)/ 0.08 (0.16)<br>Left: 11.12 (5.01) /0.07 (0.38)<br>**Eversion/inversion**<br>RMSE (°) (SD) / R (mean + SD)<br>Right: 9.13 (3.64)/ 0.17 (0.48)<br>Left: 11.3 (3.59) /-0.01 (0.27)<br>**Rotation**<br>RMSE (°) (SD) / R (mean + SD)<br>Right: 18.8 (6.67)/0.16 (0.32)<br>Left: 17.89 (7.50)/ 0.38 (0.53)
Kumar Y. & al.	19	Home-made sensors systems	Goniometer	Maximal active ROM	r^2<br>0.92(dorsiflexion)<br>0.93(plantar flexion)<br>Lower extremity ± 10°
Lebel K. & al.	20	IGS-180 (synertial) (model OSv3 also called OS3D, Inertial Labs)	Vicon Motion Systems (Oxford Metrics Ltd, Oxford, UK)	Sit-to-stand<br><br>Walking<br><br>Turning	**RMSE (°)/Err peak(°)/CMC**<br>Sit-to-stand: : 1.1 / 2.9 / 0.935<br>Walk: 2.1/ 5.7 / 0.996<br>Turn: 1.7/ 4.5 / 1.000
Mifsud NL. & al.	14	IMU<br>(Debus und Diebold Messsysteme, GmbH)	Qualisys (Medical, Gothenburg, SE)	Running (10 meter at 12 ± 1.2 km/h)	Inversion/eversion (B, frontal plan) :<br>Bias 6,1<br>LOA (95%): -6.7, 18.9<br>ICC: -0.35<br>RMSE: 6.3<br><br>Internal/external rotation (p, transverse plan) :<br>Bias: 0.3<br>LOA (95%): -15.8, 16.3<br>ICC: 0.37<br>RMS : 7,7<br><br>Horizontal sol angle (y, sagittal plan):<br>Bias: -7.1<br>LOA (95%): -18.3, 4.1<br>ICC: 0.92<br>RMSE: 7.0

Robert-Lachaine X. & al.	12	Xsens MVN BIOMECH (Xsens technologies B.V., Enschede, the Netherlands)	Optotrak system (Northern Digital Inc., Ontario, Canada)	Handling tasks and simple movements	**RMSE/CMC /LoA**<br>Z= 3.8/0.95/0.0 ±7.2<br>X= 4.3/0.89/-0.6±8.0<br>Y= 7.3/0.77/0.8 ±13.6
Rouhani, H. & al.	3	IMUs<br>(PhysilogVR , BioAGM, CH)	Vicon Motion Systems (Oxford Metrics Ltd, Oxford, UK)	Walking (2, 3.5, and 5 km/h)	**RMS differences (°)**<br>Slow walking: 1.2<br>Medium walking: 1.4<br>Fast walking :2.0<br>**Mean coefficient correlation:** 0.93<br>**Mean difference:** below 4 degrees
Saito, H. & al.	3	Wireless sensors<br>(WAA-006, Wireless Technologies)	Optotrak system (Northern Digital Inc., Ontario, Canada)	Walking (3 different speeds)	**Without Kalman Filter**<br>**RMSE (SD)**<br>5.60 (2.42)<br>***R (SD)***<br>0.768 (0.126)<br>**With Kalman Filter**<br>**RMSE (SD)**<br>3.94 (0.89)<br>***R (SD)***<br>0.824 (0.064)
Zhang, JT & al.	10	Xsens MVN BIOMECH (Xsens technologies B.V., Enschede, the Netherlands)	Optotrak system (Northern Digital Inc., Ontario, Canada)	Walking<br>Stair descent<br>Stair ascent	ROM/error (°)/CMC (only in walking)<br>**Walking:** Flexion-extension= 0.4 ± 2.58 / 2.15 ±1.24 / 0.99<br>Abduction-adduction = 1.38± 3.19/1.81±1.26 / 0.95<br>Inversion-eversion = 1.25 ± 2.37 / 1.84 ± 1.06 /0.77<br>**Stair descent:** Flexion-extension= 0.04 ± 2.56 / 3.99 ±2.05<br>Abduction-adduction = 8.06± 7.35/6.69± 3.23<br>Inversion-eversion = 0.29 ± 4.4 / 2.58 ± 1.61<br>**Stair ascent:** Flexion-extension= 0.26 ± 3.00 / 2.86 ±1.62<br>Abduction-adduction = 3.43± 4.82/ 2.56± 1.76<br>Inversion-eversion = 0.31 ± 2.93 / 1.61 ± 1.12
**Pelvis**					
Blair, S & al.	30	Xsens MVN BIOMECH (Xsens technologies B.V., Enschede, the Netherlands)	Vicon Motion Systems (Oxford Metrics Ltd, Oxford, UK)	Kicking tasks	**Mean difference ± 90% CL**<br>Australian football: 0.9 ± 0.7<br>Soccer: 0.1 ± 0.7<br>Rugby: 0.3 ± 0.7
Bolink,	17	IMUs	MOCAP, six VICON	Gait	ICC/RMSE (°)/ Pearson correlation

S. A. & al.		(MicroStrain® Inertia-Link)	MX-3+ and two VICON MX-T20 cameras	Sit-to-stand<br>Block step-up	**Gait**<br>Sagittal plane: 0.96/2.7/0.94<br>Frontal plane: 1.00/2.68/0.91<br>**Sit-to-stand**<br>Sagittal plane: 0.99/8.89/0.92<br>Frontal plane:1.00/4.44/0.89<br>**Block step-up**<br>Sagittal plane: 0.98/6.61/0.86<br>Frontal plane: 0.96/3.05/0.85
Bugane, F. & al.	16	Free4Act (F4A – LetSense Srl, Bologna, Italy)	Vicon Motion Systems (Oxford Metrics Ltd, Oxford, UK)	Walking	**Tilt**<br>**RMSE (SD)**: 0.73 (0.38)<br>**R (SD)** :0.88 (0.04)<br>**difference (SD):**<br>0.12 (0.13)<br>**Obliquity**<br>**RMSE (SD):** 1.22 (0.41)<br>**R (SD):** 0.95 (0.02)<br>**Difference ° (SD)**: 0.85 (0.46)<br>**Rotation**<br>**RMSE (SD):** 2.66 (1.57)<br>**R (SD):** 0.91 (0.11)<br>**Difference ° (SD):** 1.76 (1.03)
Lebel K. & al.	20	IGS-180 (synertial) (model OSv3 also called OS3D, Inertial Labs)	Vicon Motion Systems (Oxford Metrics Ltd, Oxford, UK)	Sit-to-stand<br>Walking<br>Turning	**RMSD (°)/Err peak(°)/CMC**<br>Sit-to-stand: 1.0/2.9/0.997<br>Walk: 2.2/5.1/0.842<br>Turn: 1.7/4.0/1.000
Plamondon & al.	6	IMUs<br>(Micro Strain 3DM-G, Burlington)	Optotrak system (Northern Digital Inc., Ontario, Canada)	Lifting tasks<br>(Static validation and dynamic validation)	RMSE (SD) °<br>**Static validation**<br>Flexion a:<br>Static: 0.7 (0.2)<br>Slow: 0.4 (0.1)<br>Moderate: 0.4 (0.1)<br>Fast: 0.4 (0.1)<br>All speeds: 0.5 (0.2)<br>Lateral b<br>Static: 0.8 (0.1)<br>Slow: 0.6 (0.1)<br>Moderate: 0,5 (0,1)<br>Fast: 0.6 (0.1)<br>All speeds: 0.7 (0.2)<br>Torsion g<br>Static: 2.4 (0.4)<br>Slow: 1.7 (0.2)

					Moderate: 1.5 (0.1)<br>Fast: 2.2 (0.3)<br>All speeds: 2.0 (0.5)<br>**Dynamic validation**<br>Flexion a:<br>RMS = 2.0<br>95th = 5.3<br>CMC = 0.974<br>Lateral b:<br>RMS =1.8<br>95th = 4.9<br>CMC = 0.975<br>Torsion g:<br>RMS = 4.7<br>95th = 15.1<br>CMC = 0.998
Schall, M. C. & al.	6	IMU system<br><br>(I2 M Motion Tracking, Series SXT)	Vicon Motion Systems (Oxford Metrics Ltd, Oxford, UK)	Simulated milking cluster attachment task	**(RMSE° (SD))**<br>Flexion-extension: 6.2 (3.0)<br>Lateral Bending: 5.0 (2.1)
**Trunk**					
Bauer, C. M. & al.	22	IMUs<br>(Valedo_ User Manual, Hocoma AG)	Vicon Motion Systems (Oxford Metrics Ltd, Oxford, UK)	Maximal range of motion - Isolated trunk movement	**r^2 / RMSE**<br>**Thoracic:**<br>Flexion= 0.95 ± 0.04 / 5.8 ±2.0<br>Extension = 0.94 ±0.09 / 5.9 ± 3.3<br>Lateral flexion right = 0.99 ± 0.01 / 2.8 ± 1.4<br>Lateral flexion left = 0.99 ± 0.03/ 2.6 ± 2.0<br>**Lumbar:**<br>Flexion= 0.99 ± 0.01 / 4.1 ±1.8<br>Extension = 0.97 ±0.05 / 4.4 ± 2.2<br>Lateral flexion right = 0.99 ± 0.01 / 1.8 ± 0.0<br>Lateral flexion left = 0.99 ± 0.01/ 1.9 ± 1.3
Bergami ni, E.	5	FreeSense,<br>Sensorize	Vicon Motion Systems (Oxford Metrics Ltd, Oxford, UK)	Running	r=0.994 ± 0.013<br>RMSE: 3°±2°
Dowlin g, A. V. & al.	38	Inertial measurement units<br>(PhysilogVR , BioAGM, CH)	Qualisys (Medical, Gothenburg, SE)	2-drop jumps with a unilateral support landing	(**R** / accuracy °/ precision °)<br>Contact: 0.75/-4.6/6.8<br>Max stance: 0.88/-5.5/5.6<br>Difference :0.72/0.0/5.4
Godwin A. & al.	1	Xsens MVN BIOMECH (Xsens technologies B.V., Enschede, the Netherlands)	Vicon Motion Systems (Oxford Metrics Ltd, Oxford, UK)	Sweep<br>Table wash<br>Asymmetric lifting	**(mean) RMSE - Max. RMSE (°)**<br>Lumbar:<br>**Sweep:** X: 1.4 – 5.7; Y: 1.3-6.3; Z: 1,0 – 5.1

					**Table wash:** X: 4.1 – 12.5; Y:3.5- 10.5; Z: 2.0 – 8,1<br>**Asymetric lifting:**<br>X: 5.5 – 12.9; Y: 9,8 – 26.3; Z: 11.2 – 26.2<br>Upper trunk:<br>**Sweep:** X: 2.3 – 5.8; Y: 1.5 – 4.6; Z: 3.8 – 10.3<br>**Table wash:** X: 4.0 – 12.1; Y: 3.4 – 7.7; Z: 6.0 – 17.4<br>**Asymetric lifting:** X: 4.9 – 15.4; Y: 6.9 – 17.1; Z: 12.4 – 32.1
Ha TH. & al.	26	Xsens MVN BIOMECH (Xsens technologies B.V., Enschede, the Netherlands)	Fastrak system (Polhemus, 2007)	Simple trunk movement	**Correlation coefficient**<br>**Flexion:** X= 0.0667 ; Y=0.8876 ; Z=0.3065<br>**Extension:** X=0.1930; Y=0.6573; Z=0.2389<br>**Right lateral flexion:** X=0.2251; Y=0.4941; Z=0.8535<br>**Left lateral flexion:** X=0.597; Y=0.7832; Z=0.900<br>**Right rotation:** X=0.6657; Y=0.5963; Z=0.2771<br>**Left rotation:** X=0.5411; Y=0.2674; Z=0.4622
Leardini A. & al.	17	Riablo™ (CoRehab, Trento, Italy)<br><br>*An adaptive system, comprised of several IMU*	Vicon Motion Systems (Oxford Metrics Ltd, Oxford, UK)	1. Lunge<br><br>2. Squat	**RMSE : Mean ± SD (°)**<br><br>1. 1.6 ± 0.6<br>2. 2.7 ± 2.1
Lebel K. & al.	20	IGS-180 (synertial) (model OSv3 also called OS3D, Inertial Labs)	Vicon Motion Systems (Oxford Metrics Ltd, Oxford, UK)	Sit-to-stand<br><br>Walking<br><br>Turning	**RMSD (°)/Err peak (°)/CMC**<br>Sit-to-stand: 1.5 /3.7/0.996<br>Walk: 2.9 /6.8 /0.853<br>Turn: 3.1 /5.6 /0.999
Mjosund HL. & al	14	ViMove	Vicon Motion Systems (Oxford Metrics Ltd, Oxford, UK)	Simple trunk movements	**RMSE ± SD/ Mean difference/LoA**<br><br>Flexion: 1.82 ±1.00/0.42/-3.86<br>Extension: 0.71 ± 0.34 / -0.12/ -2.15<br>Lateral Right: 0.77 ± 0.24/ -0.16/-2.37<br>Lateral left: 0.98 ± 0.69/ -0.08/-3.11
Plamondon & al.	6	IMUs<br>(Micro Strain 3DM-G, Burlington)	Optotrak system (Northern Digital Inc., Ontario, Canada)	Lifting task<br>(Static validation and dynamic validation)	**Mean RMSE (SD) °**<br>**Static validation**<br>Flexion a:<br>Static: 0.9 (0.1)<br>Slow: 0.7 (0.2)<br>Moderate: 0.7 (0.1)<br>Fast: 0.6 (0.1)<br>All speeds: 0.8 (0.2)<br>Lateral b<br>Static: 0.8 (0.2)<br>Slow: 0.8 (0.2)

					Moderate: 0.7 (0.1)<br>Fast: 0.6 (0.1)<br>All speeds: 0.7 (0.1)<br><u>Torsion g</u><br>Static: 24 (0.5)<br>Slow: 1.4 (0.3)<br>Moderate: 2.1 (0.3)<br>Fast: 1.4 (0.3)<br>All speeds: 1.9 (0.6)<br>**Dynamic validation**<br><u>Flexion a:</u><br>RMS =2.7<br>95th = 8.0<br>CMC = 0.993<br><u>Lateral b:</u><br>RMS = 1.9<br>95th = 6.3<br>CMC = 0.971<br><u>Torsion g:</u><br>RMS = 5.2<br>95th = 20.5<br>CMC = 0.988
Robert-Lachaine X. & al.	12	Xsens MVN BIOMECH (Xsens technologies B.V., Enschede, the Netherlands)	Optotrak system (Northern Digital Inc., Ontario, Canada)	Handling tasks and simple movements	**RMSE/CMC /LoA**<br>Z= 1.3/1.00/ 0.1 ±2.3<br>X- 1.5/0.98/-0.4±2.2<br>Y= 3.6/0.97/-2.6 ±4.5
Schall, M. C. & al.	6	IMU<br>system<br><br>(I2 M Motion Tracking, Series SXT)	Vicon Motion Systems (Oxford Metrics Ltd, Oxford, UK)	Simulated milking cluster attachment task	**<u>(RMSE° (SD))</u>**<br>Flexion-extension: 5.4 (2.6)<br>Lateral Bending: 5.8 (2.9)
**Shoulder**					
Barraza Madrigal, J. A. & al.	10	Invensense-MPU6050	Ariel Performance Analysis System (APAS)	Maximal active ROM<br><br>(Flexion Extension)	**RMSE (°)**<br><u>Max values :</u><br>X axis: 1.57<br>Y axis: 0.63<br>Z axis: 1.25<br><u>Min. Values</u> :<br>X axis: 1.13<br>Y axis: 1.31<br>Z axis: 1.98
Bouvier, B. & al.	10	Xsens MVN BIOMECH (Xsens technologies B.V., Enschede, the Netherlands)	Eagle 4 (Motion Analysis Corporation, CA, USA)	Maximal active ROM<br><br>Wheel test	**CMC / RMSE (°)**<br><u>Shoulder flexion</u><br>flexion=0.98/13.4

					internal/external rotation= 0.60/14<br>Shoulder abduction<br>flexion = 0.97/19.3<br><br>external/internal rotation = 0.83/21.8<br>Wheel<br>flexion= 0.91/14.7<br><br>abduction= 0.73/11.4<br><br>internal/external rotation = 0.74/18.1
Cutti, A. G. & al.	1	IMMS MT9B (Xsens technologies B.V., Enschede, the Netherlands)	Vicon Motion Systems (Oxford Metrics Ltd, Oxford, UK)	Maximal active ROM	**RMS error (°) /r/ *m* (angular coefficient of regression**<br><br>97% of data pairs: ranged 0.2 to 3.2/ 90.4% of data pair: above 0.86/ 88% of data pairs: 0.79 to 1.15
Ertzgaard, P. & al.	10	IMUs<br>(Analog devices, Adis 16350)	Codamotion (Charnwood Dynamics, Leicestershire, UK)	Lifting and dropping<br><br>Throwing<br><br>Task 1: Top of the head, to the shoulder, clapping back of hands together, moved hands to the knee and then to the toe<br><br>Task 2: Ears, to the eyes and then to the mouth	**ICC**<br>Cone: flexion-extension=0.952/Abduction-Adduction= 0.879/External-internal rotation= 0.943<br><br>Throwing: flexion-extension=0.975/Abduction-Adduction= 0.817/External-internal rotation= 0.905<br><br>Task 1: flexion-extension=0.902/Abduction-Adduction= 0.949/External-internal rotation= 0.799<br><br>Task 2: flexion-extension=0.943/Abduction-Adduction= 0.946/External-internal rotation= 0.768
Fantozzi, S. & al.	8	IMMU system (Opal, APDM,<br>7 units, 128 Hz, Motion Studio software beta version 1.0.0.201310221707)	SMART-DX 7000 (BTS Bioengineering, 7 cameras, 250 Hz, software version 1.10.451.0)	Swimming<br><br>(Simulated front-crawl and breaststroke)	CMC/RMSE (°)/r<br>**Front-crawl**<br>**Flex-ext:** 0.99/ 5/ 0.99<br><br>**ABD-ADD:** 0.97/10/0.95<br><br>**Int-ext rot:** 0.99/7/0.98

					**Breast-stroke:**<br>**ABD-ADD:** 0.99/ 5/1<br><br>**Int-ext rot:** 0.99/3/0.99
Gil-Agudo, A. & al.	1	Xsens MVN BIOMECH (Xsens technologies B.V., Enschede, the Netherlands)	Codamotion (Charnwood Dynamics, Leicestershire, UK)	Drinking	Flexion- extension: 0.76 ±4.04/0.998<br>Abduction- Adduction: 0.69 ±10.47 / 0.991<br>Internal- external rotation: -0.65± 5.67/ 0.992
Godwin A. & al.	1	Xsens MVN BIOMECH (Xsens technologies B.V., Enschede, the Netherlands)	Vicon Motion Systems (Oxford Metrics Ltd, Oxford, UK)	Sweep<br>Table wash<br>Asymmetric lifting	**(mean) RMSE - Max. RMSE (°)**<br>**Sweep:**<br>**Right :**X: 12.7 – 34.1; Y:8.9 – 30.8; Z:25.5 – 53.9<br>**Left:** X: 2.8 – 11.5; Y: 3.3- 13.3 ; Z: 2.8 – 10.9<br>**Table wash:**<br>**Right :** X: 14.8 – 45.8; Y: 12.4 – 39.5; Z: 25.6 – 64.1<br>**Left :** X: 7.1 – 31.8; Y: 9.3 – 32.3; Z: 12.1 – 64.5<br>**Asymmetric lifting:**<br>**Right:** X: 9.6 – 33.1; Y:13.1 – 33.2; Z: 17.1 – 55.5<br>**Left:** X: 12.9 – 49.8; Y: 16.2 – 42.2; Z: 23.6 – 74.2
Kumar Y. & al.	19	Home-made sensors systems	Goniometer	Maximum range of motion	r^2<br>0.79 to 0.89 (flexion= 0.84, extension= 0.84, abduction= 0.86, internal rotation = 0.79, external rotation = 0.89)<br>Upper extremity ± 20°
Pérez R. & al.	1	MTi Xsens inertial<br>(Xsens technologies B.V., Enschede, the Netherlands)	SMART-DX 7000 (BTS Bioengineering, 7 cameras, 250 Hz, software version 1.10.451.0)	Flexion-ext<br><br>ABD-ADD<br><br>Internal rotation<br><br>Serving water from a jar	**Correlation coefficient:**<br>Flexion-Ext: 0.994<br>ABD-ADD: 0.718<br>Inter. Rot: 0.995<br>Serving water from a jar:<br>Flexion-Ext: 0.995<br>ABD-ADD: 0.908<br>Inter. Rot: 0.853<br>**Mean peak diff. °:**<br>Flexion-Ext: 13.4<br>ABD-ADD: 17.25<br>Inter. Rot: 60.45<br>Serving water from a jar:<br>Flexion-Ext: 13.82<br>ABD-ADD: 7.44<br>Inter. Rot: 28.88
Robert-Lachaine X. & al.	12	Xsens MVN BIOMECH (Xsens technologies B.V., Enschede, the Netherlands)	Optotrak system (Northern Digital Inc., Ontario, Canada)	Handling tasks and simple movements	**RMSE/CMC/LoA**<br>X= 2.9/1.00/-0.1± 5.2<br>Z= 3.0/0.99/ -0.9±4.2<br>Y=2.5/0.99/-0.7± 3.8

Schall, M. C. & al.	6	IMU system<br><br>(I2 M Motion Tracking, Series SXT)	Vicon Motion Systems (Oxford Metrics Ltd, Oxford, UK)	Simulated milking cluster attachment task	**(RMSE° (SD))**<br>7.2-12.1 (2.4-3.2)
Zhou, H. & al.	8	MTx inertial sensors (Xsens technologies B.V., Enschede, the Netherlands)	Qualisys (Medical, Gothenburg, SE)	Activity of daily living and rotation of upper extremity at different speeds (following circle and rectangle)	**Mean ±SD (°)/RMS (°) /Correlation**<br>0.05 m/s: 1.79±1.45/2.04/0.96-<br>0.1 m/s: -2.29±2.44/2.38/0.97<br>0.15 m/s: 2.69±2.35/2.06/0.94<br>0.25 m/s: -2.13±2.03/2.05/0.98
**Elbow**					
Bouvier, B. & al.	10	MTx<br>(Xsens Technologies B. V.)	Eagle 4 (Motion Analysis Corporation, CA, USA)	Flexion<br>Extension<br>Pronation<br>Supination<br>Wheel test	**CMC / RMSE (°)**<br>flexion-extensions = 0.93/24<br>pronation-supination= 0.95/14,2<br>wheel= 0.80/24,9
Cutti, A. G. & al.	1	IMMS MT9B<br>(Xsens technologies B.V., Enschede, the Netherlands)	Vicon Motion Systems (Oxford Metrics Ltd, Oxford, UK)	Simple movement	**RMS error (°) /r/ *m* (angular coefficient of regression**<br><br>97% of data pairs : ranged 0.2 to 3.2/ 90.4% of data pair: above 0.86/ 88% of data pairs: 0.79 to 1.15
Ertzgaard, P. & al.	10	IMUs<br>(Analog devices, Adis 16350)	Codamotion (Charnwood Dynamics, Leicestershire, UK)	Lifting and dropping<br><br>Throwing<br><br>Task 1: Top of the head, to the shoulder, clapping back of hands together, moved hands to the knee and then to the toe<br><br>Task 2: Ears, to the eyes and then to the mouth	**ICC**<br>Cone: flexion- extension=0.984, pronation-supination= 0.682<br><br>Throwing: flexion- extension=0.845, pronation-supination= 0.783<br><br>Task 1: flexion- extension=0.914, pronation-supination= 0.792<br><br>Task 2: flexion- extension=0.888, pronation-supination= 0.808
Fantozzi, S. & al.	8	IMMU system (Opal, APDM,<br>7 units, 128 Hz, Motion Studio software beta version 1.0.0.201310221707)	SMART-DX 7000 (BTS Bioengineering, 7 cameras, 250 Hz, software version 1.10.451.0)	Swimming (simulated front-crawl<br>and breaststroke)	CMC/RMSE (°)/r<br><br>**Front-crawl**<br>**Flex-ext:** 0.95/15/0.96<br><br>**Pro-sup :**0.93/10/0.92<br><br>**Breast-stroke:**<br>**Flex-ext:** 0.98/8/0.99

					**Pro-sup:** 0.97/6/0.99
Gil-Agudo, A. & al.	1	Xsens MTx<br>(Xsens technologies B.V., Enschede, the Netherlands)	Codamotion (Charnwood Dynamics, Leicestershire, UK)	Drinking	Flexion-extension: -0.54 ±2.63/0.999<br>Pronation- Supination: -5.16 ±4.5/ 0.991
Godwin A. & al.	1	Xsens MTx (Xsens Technologies, Enschede, The Netherlands)	Vicon Motion Systems (Oxford Metrics Ltd, Oxford, UK)	Sweep<br>Table wash<br>Asymmetric lifting	**(mean) RMSE - Max. RMSE (°)**<br>**Sweep:**<br>**Right :**X: 8.6 – 19.4**;** Y: 2.2 – 6.5**;** Z: 5.6 – 19.3<br>**Left:** X: 1.0 – 5.8**;** Y: 0.6 – 2.5**;** Z: 1.8 – 7.0<br>**Table wash:**<br>**Right:** X: 9.7 – 30.6; Y:6.3 – 19.2**;** Z: 13.4 – 31.8<br>**Left:** X: 4.5 – 17.6**;** Y: 3.5 – 14.6**;** Z: 2.9 – 14.1
Kumar Y. & al.	19	Home-made sensors systems	Goniometer	Maximum range of motion	r^2<br>0.77 (flexion only)<br>Upper extremity ± 20°
Pérez R. & al.	1	MTi Xsens inertial<br>(Xsens technologies B.V., Enschede, the Netherlands)	SMART-DX 7000 (BTS Bioengineering, 7 cameras, 250 Hz, software version 1.10.451.0)	Flexion<br>Extension<br>Pronation<br>Supination<br>Serving water from a jar	**Correlation coefficient:**<br>Flex-ext: 0.984<br>Pro-Sup: 0.968<br>Serving water from a jar:<br>Flex-ext: 0.979<br>Pro-sup: 0.925<br>**Mean peak diff. °:**<br>Flex-ext: 5.8<br>Pro-Sup: 24.1<br>Serving water from a jar:<br>Flex-ext: 18.6<br>pro-sup: 11.7
Robert-Lachaine X. & al.	12	Xsens system<br>(Xsens technologies B.V., Enschede, the Netherlands)	Optotrak system (Northern Digital Inc., Ontario, Canada)	Handling tasks and simple movements	**RMSE/CMC/LoA**<br>Z= 2.9/0.99/0.3±5.2<br>X=2.0/0.98/0.3±3.7<br>Y= 2.6/0.99/0.6 ±4.8
Zhou, H. & al.	8	MTx inertial sensors (Xsens technologies B.V., Enschede, the Netherlands)	Qualisys (Medical, Gothenburg, SE)	Activity of daily living and rotation of upper extremity (following circle and rectangle)	**Mean ±SD (°)/RMS (°) /Correlation**<br>0.05 m/s: -2.43±1.97/2.33/0.97<br>0.1 m/s: -2.53±2.35/2.41/0.96<br>0.15 m/s: -2.11±2.67/2.25/0.97<br>0.25 m/s: -2.23±2.14/2.16/0.97
**Wrist**					
Bouvier, B. & al.	10	Xsens MTx<br>(Xsens technologies B.V., Enschede, the Netherlands)	Eagle 4 (Motion Analysis Corporation, CA, USA)	Flexion<br>Extension<br><br>Abduction	**CMC / RMSE (°)**<br>flexion-extensions :0.95 / 11.6<br>abduction-adductions :0.81/12.3

				Adduction	
Fantozzi, S. & al.	8	IMMU system (Opal, APDM,<br>7 units, 128 Hz, Motion Studio software beta version 1.0.0.201310221707)	SMART-DX 7000 (BTS Bioengineering, 7 cameras, 250 Hz, software version 1.10.451.0)	Swimming<br>(simulated front-crawl and breaststroke)	CMC/RMSE (°)/r<br>**Front-crawl**<br>**Flex-ext:** 0.95/5/0.96<br><br>**RA-UL:** 0.90/3/0.91<br><br>**Flex-ext:** 0.99/6/1<br><br>**Breast-stroke:**<br>**Flex-ext:** 0.98/5/0.98<br><br>**RA-UL:** 0.93/4/0.93
Gil-Agudo, A. & al.	1	Xsens MTx<br>(Xsens technologies B.V., Enschede, the Netherlands)	Codamotion (Charnwood Dynamics, Leicestershire, UK)	Drinking	Flexion- extension :3.47 ± 9.43/0.974<br>Radial-ulnar deviation : -2.19 ± 4.64/ 0.954
Kumar Y. & al.	19	Home-made sensors systems	Goniometer	Maximum range of motion	r^2<br>0.75 (pronation)<br>0.73(supination)<br>0.62 to 0.88(flexion=0.86, extension=0.68, radial deviation=0.88, ulnar deviation=0.62)<br><br>Upper extremity ± 20°(except for extension and ulnar deviation = ±30°)
Pérez R. & al.	1	MTi Xsens inertial (Xsens technologies B.V., Enschede, the Netherlands)	SMART-DX 7000 (BTS Bioengineering, 7 cameras, 250 Hz, software version 1.10.451.0)	Flexion<br>Extension<br>Serving water from a jar	**Correlation coefficient:**<br>Flex- ext: 0.987<br>Serving water from a jar: 0.924<br>**Mean peak diff. °:**<br>Flex- ext: 11.65<br>Serving water from a jar: 26.88
Robert-Lachaine X. & al.	12	Xsens system<br>(Xsens technologies B.V., Enschede, the Netherlands)	Optotrak system (Northern Digital Inc., Ontario, Canada)	Handling tasks and simple movements	**RMSE/CMC/LoA**<br>Z = 3.8/0.96/-1.0±6.9<br>X= 2.8/0.95/ -0.4 ±5.1<br>Y=3.6/0.92/-1.3±5.9
**Neck**					
Gil-Agudo, A. & al.	1	Xsens MTx<br>(Xsens technologies B.V., Enschede, the Netherlands)	Codamotion (Charnwood Dynamics, Leicestershire, UK)	Drinking	Flexion-extension: 1.58±1.34/ 0.999<br>Inclination: -8.24 ± 2.10 / 0.993
Godwin A. & al.	1	Xsens MTx (Xsens Technologies, Enschede, The Netherlands)	Vicon Motion Systems (Oxford Metrics Ltd, Oxford, UK)	Sweep<br>Table wash<br>Asymmetric lifting	**(mean) RMSE - Max. RMSE (°)**<br>**Sweep:** X: 1.7 – 5.2; Y: 1.3 – 5.6**;** Z:0.7-3.5<br>**Table wash:** X: 2.2 -12.8; Y: 3.0 – 11.0; Z: 9.8 –

					40.6<br>**Asymmetric lifting:** X: 11.7 – 30.9; Y: 6.3 – 18.4; Z: 14.4 – 47.1
Jasiewic, J.M et al.	10	IC3 (Intersense, Bedford, MA, USA)	Fastrak system (Polhemus, 2007)	Maximum range of motion (Left side)<br>Rotation<br>Flexion<br>Lateral flexion (left)	**Mean error – SD (°)/ RMSE – SD/ cross-correlation**<br><br>**Head mounted sensors**<br>Flexion: -0.2 – 0.8/ 2.1 – 1.1/ 0.98<br>Lateral flexion: 2.2 – 0.8/ 2.5 – 0.9/ -0.97<br>Rotation: 0.8 – 1.3/2.3 – 0.9/0.97<br>**C7/Trunk mounted sensors**<br>Flexion: -0.0 – 0.6/1.2 – 0.5/0.98<br>Lateral flexion: 0.4 – 0.3/0.7 – 0.7/0.99<br>Rotation: -1.9 – 0.7/0.9-0.5/0.98
Lebel K. & al.	20	IGS-180 (synertial) (model OSv3 also called OS3D, Inertial Labs)	Vicon Motion Systems (Oxford Metrics Ltd, Oxford, UK)	Sit-to-stand<br><br>Walking<br><br>Turning	**RMSD (°)/Err peak (°)/CMC**<br>Sit-to-stand: 4.0 /8.0/0.9<br>Walk: 3.7 /8.9/0.819<br>Turn: 4.4 /6.8/0.998
Robert-Lachaine X. & al.	12	Xsens system<br>(Xsens technologies B.V., Enschede, the Netherlands)	Optotrak system (Northern Digital Inc., Ontario, Canada)	Handling tasks and simple movements	**RMSE/CMC/LoA**<br>Z= 1.4/1.00/0.1±2.8<br>X= 1.5/1.00/-0.0±2.6<br>Y= 3.0/0.99/-1.4±5.0

RMSE: root mean square error, CMC: coefficient of multiple correlation, LoA: Limits of Agreement, Err: Error, SD: Standard deviation, Diff: Difference, Flex: flexion, Ext: extension, Abd: abduction, Add: Adduction, RA-UL: Radio-ulnar, Pro-sup: Pro-supination; ICC: intraclass correlation coefficient, ROM: Range of motion.

Table S3 : Synthesis of reliability results.

Authors	n	IMU used	Task	Reliability
**Knee**				
Al-Amri & al.	26	Xsens MVN BIOMECH (Xsens technologies B.V., Enschede, the Netherlands)	Walking<br>Jump<br>Squat	**Intra-rater reliability**<br>• Sagittal plan : ICC : 0.60 à 0.95<br>• Absolute : SEM < 5°<br>**Inter-rater reliability**<br>• Walking and squatting: ICC > 0.6 et SEM < 5°<br>• Jumping: poor to excellent
Favre, J. & al.	10	IMUs (ADXRS and ADXL) (PhysilogVR , BioAGM, CH)	Maximal active ROM<br>Walking (30m)	**Intra-rater reliability**<br>Repeatability of the θ angle (R) was 2.8°
Schiefer, C. & al	20	CUELA system (CUELA, IFA, Sankt Augustin,Germany)	Passive maximal ROM	**Intra-rater reliability (/ICC)**<br>Knee ext: 0.61<br>Knee flex: 0.82(left), 0.84 (right)
**Hip**				
Al-Amri & al.	26	Xsens MVN BIOMECH (Xsens technologies B.V., Enschede, the Netherlands)	Walking<br>Jump<br>Squat	**Intra-rater reliability**<br>• Sagittal plan : ICC : 0.60 à 0.95<br>• Absolute : SEM < 5°<br>**Inter-rater reliability**<br>• Walking and squatting: ICC > 0,6 et SEM < 5°<br>• Jumping: poor to excellent
Bauer, C. M. & al.	24	IMUs (Valedo_ User Manual, Hocoma AG)	Maximal active ROM	**Intra-rater reliability**<br>• Index of dependability: 0.19 to 0.9 (High to very reliable)
Schiefer, C. & al	20	CUELA system (CUELA, IFA, Sankt Augustin,Germany)	Maximal passive ROM	**Intra-rater reliability (ICC)**<br>Hip flex: 0.87(left), 0.92 (right)<br>Hip lat. Rot: 0.87 (left), 0.96 (right)<br>Hip med Rot: 0.91 (left), 0.93 (right)
**Ankle**				
Akins, J. S. & al.	12	MARG sensors (X-IMU, x-io Technologies Limited, UK).	Soccer field maneuvers	**Intra-rater reliability (ICC/ /SEM) *:**<br>Plantar-dorsiflexion = 0.284-0.755/2.6-7.1<br>Eversion-inversion = 0.482-0.741/1.5-3.4<br>Internal-external rotation =0.558-0.788/3.3-5.9<br><br>***only initial contact data are reported, for more details refer to article**

Al-Amri & al.	26	Xsens MVN BIOMECH (Xsens technologies B.V., Enschede, the Netherlands)	Walking<br><br>Jump<br><br>Squat	**Intra-rater reliability**<br>• Sagittal plan: ICC : 0.60 à 0.95<br>• Absolute: SEM < 5°<br>**Inter-rater reliability**<br>• Walking and squatting: ICC > 0.6 et SEM < 5°<br>• Jumping: poor to excellent
Mifsud NL. & al.	14	IMU<br>(Debus und Diebold Messsysteme, GmbH)	Running (10 meter at 12 ± 1.2 km/h)	**Reliability (test re-test/intra-rater reliability)**<br>Inversion/eversion (B, frontal plan):<br>Bias: -0.3<br>LOA (95%): -7.2, 6.6<br>ICC: 0.84<br>RMSE: 3.3<br><br>Internal/external rotation (p, transverse plan):<br>Bias: -0.4<br>LOA (95%): -10.6, 9.9<br>ICC: 0.88<br>RMSE: 5.0<br><br>Horizontal sol angle (y, sagittal plan):<br>Bias: -0.9<br>LOA (95%): -8.0, 6.2<br>ICC: 0.95<br>RMSE: 3.5
**Pelvis**				
Bugane, F. & al.	1	Free4Act (F4A – LetSense Srl, Bologna, Italy)	Walking	<u>**Intra-subject/intra-raters reliability**</u><br>**Tilt**<br>Good reliability with<br>SD: 0.3°<br>**Obliquity**<br>Good reliability with<br>SD: 0.6°<br>**Rotation**<br>Good reliability with<br>SD: 1.1°
**Trunk**				
Bauer, C. M. & al.	24	IMUs<br>(Valedo_ User Manual, Hocoma AG)	Maximal active ROM	**Intra-rater reliability**<br>• Index of dependability: 0.19 to 0.9 (High to very reliable [except for the extension of lumbar spine])
Schiefer, C. & al	20	CUELA system<br>(CUELA, IFA, Sankt Augustin,Germany)	Maximal active ROM	**Intra-rater reliability (ICC)**<br>Thoracic and lumbar rotation: 0.8(left), 0.9 (right)<br>Thoracic dans lumbar lat flex: 0.9 (left), 0.92 (right)

Yun, W.S. & al.	19	MEMS-IMU transmitters (EBIMU24G, E2BOX, Seoul, South Korea)	Maximal active ROM	**Intra-rater reliability (ICC)**<br>**Intra-test reliability**<br>**(first/second session)**<br><u>Sagittal</u><br>Lateral bending R: 0.983/0.958<br>Lateral bending L: 0.947/0.945<br>Rotation R: 0.975/0.949<br>Rotation L: 0.896/0.944<br><u>Coronal</u><br>Flexion: 0.996/0.997<br>Extension: 0.983/0.958<br>Rotation R: 0.918/0.965<br>Rotation L: 0.966/0.916<br><u>Axial</u><br>Flexion: 0.992/0.989<br>Extension: 0.974/0.988<br>Lateral bending R: 0.975/0.965<br>Lateral bending L: 0.982/0.989<br><br>**Test–retest reliability**<br><u>Sagittal</u><br>Lateral bending R: 0.930<br>Lateral bending L: 0.834<br>Rotation R: 0.793<br>Rotation L: 0.815<br><u>Coronal</u><br>Flexion: 0.772<br>Extension: 0.930<br>Rotation R: 0.690<br>Rotation L: 0.669<br><u>Axial</u><br>Flexion: 0.865<br>Extension: 0.862<br>Lateral bending R: 0.862<br>Lateral bending L: 0.890
**Shoulder**				
Bouvier, B. & al.	10	Xsens MVN BIOMECH (Xsens technologies B.V., Enschede, the Netherlands)	Maximal active ROM<br><br>Wheel test	**Intra-rater reliability**<br>**CMC / m (°) /** ***r***<br><u>Flexion</u><br>Flexion=0.99/5.9/1.2<br><br>Abduction= 0.72/4.2/1.6

				External/internal rotation = 0.76/6.6/2.1<br><u>Abduction</u><br>Flexion = 0.99/6.2/1.3<br><br>Abduction= 0.85/5.7/1.6<br><br>External/internal rotation = 0.91/7.5/1.8<br><u>Wheel</u><br>Flexion= 0.96/7.2/1.9<br><br>Abduction= 0.91/5.3/1.4<br><br>Internal/external rotation= 0.87/7.8/1.6
Schiefer, C. & al	20	CUELA system (CUELA, IFA, Sankt Augustin,Germany)	Maximal Passive ROM	**Intra-rater reliability (ICC)**<br>Shoulder ER: 0.71 (left), 0.86 (right)<br>Shoulder IR: 0.88 (left), 0.87 (right)
**Scapula**				
van den Noort, J. C & al.	20	MTw, (Xsens technologies B.V., Enschede, the Netherlands)	Arm elevation	**(ICC/SEM)** (scapular retraction-protraction; Scapular mediolateral rotation; Scapular anterior-posterior tilt)<br>**Interrater reliability**<br>**Anteflexion**<br><u>Upward</u><br>0°: 0.68/6;0.79/3; 0.32/2<br>30°: 0.68/6; 0.79/3; 0.39/3<br>60°: 0.68/7; 0.8/3; 0.59/3<br>90°: 0.72/6; 0.81/4; 0.73/4<br>120°:0.76/6; 0.85/4; 0.71/4<br><u>Downward:</u><br>120°: 0.77/6;0.85/4; 0.69/4<br>90°: 0.77/6; 0.76/4; 0.73/3<br>60°:0.73/6; 0.71/4; 0.66/2<br>30°: 0.71/7; 0.72/3; 0.47/2<br>0°: 0.69/7; 0.75/3; 0.41/2<br>**Abduction**<br><u>Upward</u><br>0°: 0.81/5;0.79/3; 0.56/2<br>30°: 0.68/6; 0.81/3; 0.37/2<br>60°: 0.65/6;0.87/3; 0.5/3<br>90°: 0.68/6; 0.89/3; 0.82/3<br>120°: 0.72/6; 0.89/3; 0.82/3<br>150°: 0.69/7; 0.9/3; 0.87/3<br><u>Downward:</u>

				150°:0.67/8; 0.91/3; 0.87/3<br>120°: 0.69/6; 0.88/3; 0.84/3<br>90°:0.68/6; 0.86/3; 0.79/2<br>60°: 0.66/6/; 0.82/3; 0.59/3<br>30°: 0.66/6; 0.74/3; 0.5/3<br>0°: 0.74/6; 0.76/3; 0.54/2<br><br>**Intra-raters reliability**<br>**Anteflexion**<br><u>Upward</u><br>0°: 0.83/4; 0.6/4; 0.34/2<br>30°: 0.85/4; 0.68/4; 0.39/3<br>60°: 0.83/4; 0.78/4; 0.59/3<br>90°:0.82/5; 0.86/3; 0.64/4<br>120°:0.80/5; 0.88/3; 0.67/5<br><u>Downward:</u><br>120°:0.83/5; 0.87/3; 0.68/4<br>90°:0.82/5; 0.81; 0.62/4<br>60°:0.8/5; 0.69/4; 0.57/3<br>30°:0.79/5; 0.6/4; 0.51/2<br>0°: 0.8/5; 0.59/4; 0.48/<br>**Abduction**<br><u>Upward:</u><br>0°: 0.83/4; 0.74/3, 0.78/1<br>30°: 0.82/4; 0.71/4; 0.76/1<br>60°:0.8/4; 0.79/3; 0.81/2<br>90°:0.8/4;0.84/3; 0.78/3<br>120°:0.81/4; 0.85/3; 0.75/4<br>150°: 0.78/5; 0.84/4; 0.71/5<br><u>Downward:</u><br>150°:0.78/5;0.82/4; 0.69/5<br>120°: 0.79/5; 0.83/4; 0.73/4<br>90°:0.77/4; 0.79/3; 0.71/3<br>60°:0.77/4; 0.69/4; 0.68/2<br>30°:0.78/4;0.56/4; 0.79/2<br>0°:0.78/4; 0.69/3; 0.75/2
**Elbow**				
Bouvier, B. & al.	10	Xsens MVN BIOMECH (Xsens technologies B.V., Enschede, the Netherlands)	Maximal active ROM<br><br>Wheel test	**Intra-rater reliability**<br>**CMC / m (°) / *r***<br>flexion-extensions = 0.99/6.2/1.2<br><br>pronation-supination = 0.96/7.6/2.2

				wheel = 0,94/10,0/1,2
Schiefer, C. & al	20	CUELA system (CUELA, IFA, Sankt Augustin,Germany)	Maximal passive ROM	**Intra-rater reliability (Mean SD/ICC)**<br>Elbow ext: 3.01/ 0.2 (left), 0.59 (right)<br>Elbow Flex: 4.06/ 0.69 (left), 0.77 (right)<br>Elbow pron: 4.76/ 0.48 (left), 0.43 (right)<br>Elbow sup: 3.94/ 0.39 (left), 0.7 (right)
**Wrist**				
Bouvier, B. & al.	10	Xsens MVN BIOMECH (Xsens technologies B.V., Enschede, the Netherlands)	Maximal active ROM	**Intra-rater reliability**<br>**CMC / m (°) / *r***<br><br>flexion-extensions= 0.96/9.0/1.6<br><br>abduction-adductions = 0.96/5.2/1.4
Schiefer, C. & al	20	CUELA system (CUELA, IFA, Sankt Augustin,Germany)	Maximal passive ROM	**Intra-rater reliability (Mean SD/ICC)**<br>Wrist ext: 3.58/ 0.88 (left), 0.9 (right)<br>Wrist flex: 4.23 0.9 (left), 0.86 (right)<br>Wrist ABD: 2.97/ 0.79 (left), 0.89 (right)<br>Wrist ADD: 2.93/ 0.82 (left), 0.87 (right)
**Neck**				
Duc, C. & al.	10	IMUs (Physilog®, BioAGM, CH)	Maximal active ROM	**Intra-rater reliability ICC / SEM °**<br>**Primary movement:**<br>Ax Flexion-extension (FE): 1.00/1.2<br>Ay axial rotation (AR): 0.98/3.1<br>Az lateral bending (LB): 0.87/9.4<br>**Associate movement:**<br>Ay (LB): 0.88/3.5<br>Az (LB): 0.87/2.1<br>Ax (AR): 0.75/3.7<br>Az (AR): 0.64/2.5<br>Ax (FE): 0.91/1.9<br>Ay (FE): 0.82/1.1
Kim, H. & al.	18	IMUs (model EBIMU24G, E2BOX, Seoul, Republic Korea)	Maximal active ROM	**Mean° (SD) /ICC (95% IC)**<br>**Intra-rater reliability**<br>**Flexion and extension:**<br>**Natural test**<br>Total: 116.70 ± 17.26/0.98 (0.96–0.99)<br>Extension: 57.23 ± 8.80/0.97 (0.93–0.99)<br>Flexion: 58.48 ± 11.83/0.98 (0.96–0.99)<br>**Neutral test**<br>Total (sagittal): 106.76 ± 20.33/0.99 (0.97–1.00)<br>Extension: 52.75 ± 9.25/0.95 (0.90–0.98)<br>Flexion: 54.02 ± 13.46/0.98 (0.97–0.99)

				**Rotation:**<br>**<u>Natural test</u>**<br>Total (transverse): 89.42 ± 10.1/ 0.97 (0.95–0.99)<br>Left: 44.15 ± 6.72/ 0.97 (0.93–0.99)<br>Right: 45.28 ± 4.97/ 0.98 (0.95–0.99)<br>**<u>Neutral test</u>**<br>Total (transverse): 83.69 ± 13.79/ 0.99 (0.98–1.00)<br>Left: 42.17 ± 7.33/ 0.98 (0.96–0.99)<br>Right: 41.52 ± 7.11/ 0.98 (0.97–0.99)<br>**Lateral flexion:**<br>**<u>Natural test</u>**<br>Total (transverse): 143.29 ± 17.02/ 0.99 (0.97–0.99)<br>Left: 69.67 ± 10.87/0.99 (0.97–0.99)<br>Right: 73.62 ± 10.81/0.99 (0.97–0.99)<br>**<u>Neutral test</u>**<br>Total (transverse): 134.87 ± 22.43/ 0.98 (0.97–0.99)<br>Left: 64.89 ± 11.74/ 0.98 (0.96–0.99)<br>Right: 69.98 ± 13.99/ 0.99 (0.98–1.00)
Schiefer, C. & al	20	CUELA system (CUELA, IFA, Sankt Augustin,Germany)	Maximal active ROM	**Intra-rater reliability (Mean SD/ICC)**<br>Cervical rotation: 2.6/0.79<br>Cervical extension: 2.54/ 0.83<br>Cervical flexion: 2.86/ 0.83<br>Cervical lat. flex: 1.98/ 0.93 (left), 0.95 (right)

RMSE: root mean square error, CMC: coefficient of multiple correlation, LoA: Limits of Agreement, SD: Standard deviation, Flex: flexion, Ext: extension, Pro-sup: Pro-supination; ICC: intraclass correlation coefficient, ROM: Range of motion, Standard error of the mean, lat.: lateral; l: left, R: right.

Table S4: Assessment of methodological quality by an appraisal quality tool (MacDermid et al., 2008).

Authors	Q1	Q2	Q3	Q4	Q5	Q6	Q7	Q8	Q9	Q10	Q11	Q12	Total score		
													Total	%	Agreement
**Akins, J. S. & al**	2	2	2	1	2	2	1	2	2	2	2	2	22	92	83
**Al-Amri & al.**	2	2	0	2	2	2	2	2	2	2	2	2	22	92	92
**Barraza Madrigal, J. A. & al**	1	2	0	1	0	N/A	2	2	2	1	1	2	14	64	73
**Bauer, C. M. & al.**	2	2	0	1	1	2	2	2	2	1	1	2	18	75	92
**Bergamini, E.**	1	1	0	1	0	N/A	2	1	2	2	1	2	13	59	67
**Bergmann, J. H. & al.**	1	1	0	0	0	N/A	1	2	2	2	1	2	12	55	83
**Blair, S & al.**	2	2	0	0	1	N/A	1	2	2	1	2	2	15	68	75
**Bouvier, B. & al.**	2	1	0	2	0	2	2	2	2	2	2	2	19	79	83
**Bolink, S. A. & al.**	2	2	0	0	0	N/A	2	2	2	2	1	1	14	64	92
**Bugane, F. & al.**	2	2	1	1	0	N/A	2	2	2	2	1	2	17	77	100
**Cloete, T. & al.**	2	1	0	0	0	N/A	2	2	1	2	1	2	13	59	58
**Cutti, A. G. & al.**	2	1	0	0	1	N/A	2	2	2	2	1	2	15	68	75
**Dejnabadi, H. & al.**	1	1	0	0	0	N/A	2	2	2	2	1	2	13	59	92
**Dowling, A. V. & al.**	1	2	0	1	2	N/A	2	2	2	2	1	2	17	77	92
**Duc, C. & al.**	1	2	1	2	0	N/A	2	1	2	2	2	2	17	77	67
**Ertzgaard, P. & al.**	2	2	0	1	0	N/A	1	2	2	2	1	1	14	64	83
**Fantozzi, S. & al.**	1	1	0	0	0	N/A	2	1	2	2	1	1	11	50	92
**Favre, J. & al.**	1	1	1	1	0	N/A	2	2	1	1	2	2	14	64	67
**Godwin A. & al**	2	0	0	0	0	N/A	1	1	2	2	1	2	11	50	58
**Gil-Agudo, A. & al.**	2	0	0	0	0	N/A	2	1	2	1	0	1	9	41	100
**Ha TH. & al.**	2	2	0	0	0	N/A	1	2	2	2	1	1	13	59	75
**Jasiewic, J.M et al.**	1	1	0	0	0	N/A	2	1	2	1	2	2	12	55	92
**Jaysrichai T. & al.**	1	1	0	0	0	N/A	2	1	2	2	1	1	11	50	100

**Kim & al.**	2	2	0	0	0	N/A	2	2	2	2	1	2	15	68	92
**Kumar Y. & al.**	1	1	0	0	1	N/A	1	2	2	2	2	1	13	59	83
**Leardini A. & al.**	1	1	0	1	0	N/A	2	2	2	1	1	2	13	59	83
**Lebel K. & al.**	2	1	0	1	2	N/A	2	2	2	2	2	2	18	82	83
**Mjosund HL. & al**	2	2	1	0	2	N/A	2	2	2	2	2	2	19	86	92
**Mifsud NL. & al.**	1	0	1	1	0	0	2	2	2	2	1	2	14	58	67
**Pérez R. & al.**	1	0	0	0	0	N/A	2	2	2	2	1	2	12	55	75
**Plamondon & al.**	2	1	2	0	0	N/A	2	2	2	2	2	2	17	77	67
**Robert-Lachaine X. & al.**	1	2	2	0	0	N/A	2	2	2	2	1	2	16	73	83
**Rouhani, H. & al.**	1	1	1	0	0	N/A	1	1	2	2	0	2	11	50	75
**Saber-Sheikh, K. & al.**	2	1	0	1	0	N/A	1	1	2	1	0	1	10	45	75
**Saito, H. & al.**	1	0	0	0	0	N/A	1	1	2	2	1	2	10	45	92
**Schall, M. C. & al.**	1	1	0	1	0	N/A	2	1	2	1	2	2	13	59	83
**Schiefer, C. & al**	2	2	1	2	0	2	1	1	1	1	1	2	16	67	83
**Takeda, R. & al.**	1	1	0	0	0	N/A	1	1	2	2	1	2	11	50	83
**van den Noort, J. C & al.**	2	1	1	2	0	2	2	2	2	2	1	2	19	79	83
**Yun, W.S. & al.**	2	2	1	1	0	2	2	2	2	2	1	2	19	79	92
**Zhang, JT & al.**	2	1	0	0	0	N/A	2	1	2	2	2	2	14	64	83
**Zhou, H. & al.**	1	0	0	0	0	N/A	2	2	2	2	1	1	11	50	100

Design requirements: Q1. Was the relevant background research cited to define what is currently known about the psychometric properties of the measures under study, and the need or potential contributions of the current research question? Q2. Were appropriate inclusion/exclusion criteria defined? Q3. Were specific psychometric hypotheses identified? Q4. Was an appropriate scope of psychometric properties considered? Q5. Was an appropriate sample size used? Q6. Was appropriate retention/follow-up obtained? (Studies involving retesting or follow-up only) Q7. Documentation: Were specific descriptions provided or referenced that explain the measures and its correct application/interpretation (to a standard that would allow replication)? Q8. Standardized Methods: Were administration and application of measurement techniques within the study standardized and did they are considered potential sources of error/misinterpretation? Q9. Were analyses conducted for each specific hypothesis or purpose? Q10. Were appropriate statistical tests conducted to obtain point estimates of the psychometric property? Q11. Were appropriate ancillary analyses were done to describe properties beyond the point estimates (Confidence intervals, benchmark comparisons, SEM/MID)? Q12. Were the conclusions/clinical recommendations supported by the study objectives, analysis and results?[14]. N/A: not applicable; 2= fully meet criteria; 1 = partially meet criteria; 0= not meeting criteria at all.

Table S5: Assessment of studies examining measurement errors (absolute measures) using COSMIN checklist (box B).

Authors	Q1	Q2	Q3	Q4	Q5	Q6	Q7	Q8	Q9	Q10	Q11	Q12	Q13	Q14	Total score		
															Total	%	Agreement
**Akins, J. S. & al**	1	1	1	1	1	1	N/A	1	1	1	1	N/A	N/A	N/A	10	100	100
**Al-Amri & al.**	1	1	1	1	1	1	N/A	1	1	1	1	N/A	N/A	N/A	10	100	90
**Barraza Madrigal, J. A. & al**	0	0	0	1	1	1	N/A	1	1	1	0	N/A	N/A	N/A	6	60	90
**Bauer, C. M. & al.**	0	0	1	1	1	1	N/A	1	1	1	0	N/A	N/A	N/A	7	70	100
**Bouvier, B. & al.**	0	0	0	1	0	1	N/A	1	1	1	1	N/A	N/A	N/A	6	60	90
**Bugane, F. & al.**	0	0	1	1	1	1	N/A	1	1	1	1	N/A	N/A	N/A	8	80	80
**Duc, C. & al.**	1	1	0	1	0	1	N/A	1	1	1	1	N/A	N/A	N/A	8	80	90
**Ertzgaard, P. & al.**	1	0	0	1	0	1	N/A	1	1	1	1	N/A	N/A	N/A	7	70	100
**Favre, J. & al.**	0	0	0	1	0	1	N/A	1	1	0	0	N/A	N/A	N/A	4	40	90
**Kim & al.**	0	0	0	1	1	1	N/A	1	1	0	1	N/A	N/A	N/A	6	60	80
**Mifsud NL. & al.**	1	1	0	1	1	1	N/A	1	1	1	1	N/A	N/A	N/A	9	90	100
**Schall, M. C. & al.**	0	0	0	1	1	0	N/A	N/A	0	0	0	N/A	N/A	N/A	2	22	56
**Schiefer, C. & al**	0	0	0	1	0	1	N/A	1	1	0	0	N/A	N/A	N/A	4	40	60
**van den Noort, J. C & al.**	1	1	0	1	1	1	N/A	1	1	1	1	N/A	N/A	N/A	9	90	100
**Yun, W.S. & al.**	0	0	0	1	0	1	N/A	1	1	0	1	N/A	N/A	N/A	5	50	80

Design requirements: Q1. Was the percentage of missing items given? Q2. Was there a description of how missing items were handled? Q3. Was the sample size included in the analysis adequate? Q4. Were at least two measurements available? Q5. Were the administration's independent? Q6. Was the time interval stated? Q7. Were patients stable in the interim period on the construct to be measured? Q8. Was the time interval appropriate? Q9. Were the test conditions similar for both measurements? e.g. type of administration, environment, instructions. Q10. Were there any important flaws in the design or methods of the study? Statistical methods: Q11. For continuous scores: Was an intraclass correlation coefficient (ICC) calculated? Q12. for dichotomous/nominal/ordinal scores: Was kappa calculated? Q13. For ordinal scores: Was a weighted kappa calculated? Q14. For ordinal scores: Was the weighting scheme described? e.g. linear, quadratic.[13] ; N/A: not applicable; 1 = yes; 0= no.

Table S6: Assessment of studies examining measurement errors (absolute measures) using COSMIN checklist (box C).

Authors	Q1	Q2	Q3	Q4	Q5	Q6	Q7	Q8	Q9	Q10	Q11	Total score		
												Total	%	Agreement
**Akins, J. S. & al**	1	1	1	1	1	1	N/A	1	1	1	1	10	100	80
**Al-Amri & al.**	1	1	1	1	1	1	N/A	1	1	1	1	10	100	90
**Barraza Madrigal, J. A. & al**	0	0	0	1	1	1	N/A	1	1	1	1	7	70	100
**Bergamini, E.**	0	0	0	1	1	0	N/A	N/A	1	1	1	5	56	100
**Blair, S & al.**	0	0	1	1	1	1	N/A	1	1	1	1	8	80	90
**Bouvier, B. & al.**	0	0	0	1	1	1	N/A	1	1	1	1	7	70	100
**Bolink, S. A. & al.**	0	0	0	1	1	1	N/A	1	1	1	1	7	70	100
**Bugane, F. & al.**	0	0	0	1	1	1	N/A	1	1	1	1	7	70	100
**Cutti, A. G. & al.**	0	0	0	1	1	1	N/A	1	1	1	1	7	70	100
**Dowling, A. V. & al.**	0	0	1	1	1	1	N/A	1	1	1	1	8	80	100
**Duc, C. & al.**	1	1	0	1	0	1	N/A	1	1	1	1	8	80	100
**Favre, J. & al.**	0	0	0	1	N/A	0	N/A	1	1	1	1	5	56	78
**Jaysrichai T. & al.**	0	0	0	1	1	1	N/A	1	1	1	1	7	70	100
**Kim & al.**	N/A	N/A	N/A	N/A	N/A	N/A	N/A	N/A	N/A	N/A	N/A	N/A	N/A	N/A
**Kumar Y. & al.**	0	0	0	1	1	1	N/A	1	1	1	1	7	70	100
**Leardini A. & al.**	0	0	0	1	1	1	N/A	1	1	1	1	7	70	100
**Lebel K. & al.**	0	0	1	1	0	1	N/A	1	1	1	1	7	70	90
**Pérez R. & al.**	0	0	0	1	0	1	N/A	1	1	1	1	6	60	100
**Robert-Lachaine X. & al.**	0	0	0	1	1	1	N/A	1	1	1	1	7	70	100
**Schall, M. C. & al.**	0	0	0	1	1	0	N/A	1	1	1	1	6	60	90
**Schiefer, C. & al**	0	0	0	1	1	1	N/A	1	1	1	1	7	70	80
**van den Noort, J. C & al.**	1	1	0	1	1	1	N/A	1	1	1	1	9	90	100
**Zhou, H. & al.**	0	0	0	1	0	1	N/A	1	1	1	1	6	60	100

Design requirements: Q1. Was the percentage of missing items given? Q2. Was there a description of how missing items were handled? Q3. Was the sample size included in the analysis adequate? Q4. Were at least two measurements available? Q5. Were the administration's independent? Q6. Was the time interval stated? Q7. Were patients stable in the interim period on the construct to be measured? Q8. Was the time interval appropriate? Q9. Were the test conditions similar for both measurements? e.g. type of administration, environment, instructions. Q10. Were there any important flaws in the design or methods of the study? Statistical methods: Q11. for CTT: Was the Standard Error of Measurement (SEM), Smallest Detectable Change (SDC) or Limits of Agreement (LoA) calculated? [13] ; N/A: not applicable; 1 = yes; 0= n.

Table S7: Assessment of studies examining criterion validity using COSMIN checklist (box H).

Authors	Q1	Q2	Q3	Q4	Q5	Q6	Q7	Total score		
								Total	%	Agreement
**Akins, J. S. & al**	1	1	1	1	1	1	N/A	6	100	71
**Al-Amri & al.**	1	1	1	1	1	1	N/A	6	100	100
**Barraza Madrigal, J. A. & al**	0	0	0	1	1	0	N/A	2	33	86
**Bauer, C. M. & al.**	0	0	1	1	1	1	N/A	4	67	86
**Bergamini, E.**	0	0	0	1	1	1	N/A	3	50	100
**Bergmann, J. H. & al.**	0	0	0	1	1	1	N/A	3	50	100
**Blair, S & al.**	0	0	1	1	1	0	N/A	3	50	86
**Bouvier, B. & al.**	0	0	1	1	1	1	N/A	4	67	86
**Bolink, S. A. & al.**	0	0	0	1	1	1	N/A	3	50	100
**Bugane, F. & al.**	0	0	0	1	1	1	N/A	3	50	100
**Cloete, T. & al.**	0	0	0	1	1	1	N/A	3	50	100
**Cutti, A. G. & al.**	1	0	1	1	1	1	N/A	5	83	71
**Dejnabadi, H. & al.**	1	1	0	1	1	1	N/A	5	83	71
**Dowling, A. V. & al.**	0	0	1	1	1	1	N/A	4	67	100
**Duc, C. & al.**	1	1	1	1	1	1	N/A	6	100	86
**Ertzgaard, P. & al.**	1	0	0	1	1	1	N/A	4	67	100
**Fantozzi, S. & al.**	0	0	0	1	1	1	N/A	3	50	86
**Favre, J. & al.**	0	0	0	1	1	1	N/A	3	50	100
**Godwin A. & al**	0	0	0	1	0	0	N/A	1	17	86
**Gil-Agudo, A. & al.**	0	0	0	1	0	1	N/A	2	33	100
**Ha TH. & al.**	0	0	0	1	0	1	N/A	2	33	86
**Jasiewic, J.M et al.**	0	0	0	1	1	1	N/A	3	50	86
**Jaysrichai T. & al.**	0	0	0	1	1	1	N/A	3	50	100
**Kim & al.**	0	0	0	1	1	1	N/A	3	50	100
**Kumar Y. & al.**	0	0	1	1	1	1	N/A	4	67	86
**Leardini A. & al.**	0	0	0	1	1	0	N/A	2	33	86

**Lebel K. & al.**	0	0	1	1	1	1	N/A	4	67	100
**Mjosund HL. & al**	1	0	1	1	1	1	N/A	5	83	86
**Mifsud NL. & al.**	1	1	0	1	1	1	N/A	5	83	100
**Pérez R. & al.**	0	0	0	1	1	1	N/A	3	50	100
**Plamondon & al..**	1	0	0	1	1	1	N/A	4	67	86
**Robert-Lachaine X. & al.**	0	0	0	1	1	1	N/A	3	50	86
**Rouhani, H. & al.**	0	0	0	1	1	1	N/A	3	50	100
**Saber-Sheikh, K. & al.**	0	0	0	1	1	0	N/A	2	33	100
**Saito, H. & al.**	0	0	0	1	0	1	N/A	2	33	86
**Schall, M. C. & al.**	0	0	0	1	1	0	N/A	2	33	100
**Takeda, R. & al.**	0	0	0	1	1	1	N/A	3	50	100
**Zhang, JT & al.**	0	0	0	1	1	1	N/A	3	50	100
**Zhou, H. & al.**	0	0	0	1	1	1	N/A	3	50	100

Design requirements: 1. Was the percentage of missing items given? 2. Was there a description of how missing items were handled? 3. Was the sample size included in the analysis adequate? 4. Can the criterion used or employed be considered as a reasonable 'gold standard'? 5. Were there any important flaws in the design or methods of study? Statistical methods: 6. for continuous scores: Were correlations, or the area under the receiver operating curve calculated? 7. for dichotomous scores: Were sensitivity and specificity determined?[13] ; N/A: not applicable; 1 = yes; 0= no.
